# ScopeViewer: a browser-based solution for visualizing large biological images

**DOI:** 10.1093/gigascience/giag074

**Published:** 2026-06-22

**Authors:** Danni Luo, Sophie Robertson, Yuanchun Zhan, Ruichen Rong, Shidan Wang, Xi Jiang, Sen Yang, Suzette Palmer, Peiran Quan, Hiroaki Kanzaki, Yujin Hoshida, Liwei Jia, Qiwei Li, Guanghua Xiao, Xiaowei Zhan

**Affiliations:** Quantitative Biomedical Research Center, Peter O’Donnell Jr. School of Public Health, UT Southwestern Medical Center, 5323 Harry Hines Blvd., Dallas, TX 75390-8821, USA; Paul Allen School of Computer Science & Engineering, University of Washington, 185 E Stevens Way NE, Seattle, WA 98195, USA; Quantitative Biomedical Research Center, Peter O’Donnell Jr. School of Public Health, UT Southwestern Medical Center, 5323 Harry Hines Blvd., Dallas, TX 75390-8821, USA; Quantitative Biomedical Research Center, Peter O’Donnell Jr. School of Public Health, UT Southwestern Medical Center, 5323 Harry Hines Blvd., Dallas, TX 75390-8821, USA; Quantitative Biomedical Research Center, Peter O’Donnell Jr. School of Public Health, UT Southwestern Medical Center, 5323 Harry Hines Blvd., Dallas, TX 75390-8821, USA; Quantitative Biomedical Research Center, Peter O’Donnell Jr. School of Public Health, UT Southwestern Medical Center, 5323 Harry Hines Blvd., Dallas, TX 75390-8821, USA; Quantitative Biomedical Research Center, Peter O’Donnell Jr. School of Public Health, UT Southwestern Medical Center, 5323 Harry Hines Blvd., Dallas, TX 75390-8821, USA; Quantitative Biomedical Research Center, Peter O’Donnell Jr. School of Public Health, UT Southwestern Medical Center, 5323 Harry Hines Blvd., Dallas, TX 75390-8821, USA; Quantitative Biomedical Research Center, Peter O’Donnell Jr. School of Public Health, UT Southwestern Medical Center, 5323 Harry Hines Blvd., Dallas, TX 75390-8821, USA; Department of Internal Medicine, UT Southwestern Medical Center, 5323 Harry Hines Blvd., Dallas, TX 75390-9030, USA; Department of Internal Medicine, UT Southwestern Medical Center, 5323 Harry Hines Blvd., Dallas, TX 75390-9030, USA; Department of Pathology, University of Texas Southwestern Medical Center, 5323 Harry Hines Blvd., Dallas, TX 75390-9072, USA; Department of Mathematics Sciences, The University of Texas at Dallas Mathematical Sciences, FO35 800 W. Campbell Road Richardson, TX 75080-3021, USA; Quantitative Biomedical Research Center, Peter O’Donnell Jr. School of Public Health, UT Southwestern Medical Center, 5323 Harry Hines Blvd., Dallas, TX 75390-8821, USA; Quantitative Biomedical Research Center, Peter O’Donnell Jr. School of Public Health, UT Southwestern Medical Center, 5323 Harry Hines Blvd., Dallas, TX 75390-8821, USA

**Keywords:** Spatial transcriptomics, Digital pathology, Web-based visualization, Tumor microenvironment, Gene expression profiling, H&E staining, Data privacy, MASLD

## Abstract

**Background:**

Spatial transcriptomics (ST) enables a high-resolution interrogation of molecular characteristics within specific spatial contexts and tissue morphology. Despite its potential, visualization of ST data is a challenging task due to the complexities in handling, sharing, and visualizing large image datasets together with molecular information.

**Results:**

We introduce ScopeViewer, a browser-based software designed to overcome these challenges. ScopeViewer offers the following functionalities: (1) it visualizes large image data and associated annotations at various zoom levels, allowing for intricate exploration of the data; (2) it enables dual interactive viewing of the original images along with their annotations, providing a comprehensive understanding of the context; (3) it displays spatial molecular features with optimized bandwidth, ensuring a smooth user experience; and (4) it bolsters data security by circumventing data transfers.

**Conclusions and discussions:**

ScopeViewer offers the research community a convenient, powerful, and secure software for high-resolution images, including pathology images and ST. It serves as an open-source platform for imaging-based research. Future enhancements and new features will be shared on GitHub by the creators and are open for contributions from other researchers. ScopeViewer is freely available on the website at https://cdc.biohpc.swmed.edu/scopeviewer.

## Introduction

Spatial transcriptomics (ST) technologies have made significant advancements in recent years [1]. ST techniques offer high-resolution transcriptome measurements with spatial information within tissues, thereby opening new avenues for understanding cellular and molecular spatial distributions [2] and their associated links to diseases [3]. Recent computational advances have focused on improving ST analysis through smoothing, spatial-domain identification, and graph-based representation learning approaches, including EAGS [4], Siamese graph autoencoders [5], and graph-attention autoencoder frameworks [6]. A typical ST dataset pairs with high-resolution images (e.g., H&E pathology slides), often comprising millions of pixels. This facilitates a dual visualization of cellular and tissue structures alongside quantitative molecular features, including gene expression and protein abundance. Examining molecular characteristics within spatial and morphological contexts could pave the way for new biological discoveries. A comprehensive tool for visualizing ST data will streamline data exploration and analysis, aiding researchers in comprehending molecular features within specific biological contexts.

Working with high-resolution tissue images and ST data introduces significant challenges due to the huge volume of these datasets. Consider a standard pathology image of 20,000 by 20,000 pixels, 0.5 μm per pixel, which can amass a file size of ~1 GB. This large size complicates both the image’s transfer and visualization, often requiring specialized software tools. Moreover, there is a high degree of complexity inherent in visualizing high-dimensional molecular features alongside the intricate cell and tissue structures. As a result, many software packages [7–9] currently require specific preprocessing steps to display molecular details alongside high-resolution tissue images simultaneously. Further complications arise from software interfaces that require tedious manual input from users to toggle the visibility of data layers. A more streamlined solution is a synchronized dual-view approach, which addresses the limitations of single-view interfaces where molecular overlays often obscure underlying tissue morphology. This would allow users to see the data layer in one view, while simultaneously hiding it in another, with synchronized panning and zooming capabilities. Lastly, the ability for researchers to explore ST data on their own systems, without uploading or sharing data externally, is an important consideration. This not only bolsters data security but also enhances user accessibility. To address these prevalent challenges, we developed ScopeViewer, a browser-based visualization software, available online [10]. A Docker image is also freely available online [11].

## Methods and results

ScopeViewer operates as a web application, requiring nothing more than a web browser for its execution. It was designed using the ReactJS JavaScript framework. To utilize ScopeViewer, users simply navigate to the website and input the image information and ST data from a local path, using the JSON syntax. ScopeViewer generates an interactive user interface directly within the web browser. The platform’s design leverages the versatility of web browsers, thereby eliminating the need for users to install specific software on their hardware.

### Support for multiple imaging formats

When conducting pathology image analysis or exploring ST data, it is crucial to view the image at varying magnification levels. Additionally, users often need to overlay various sources for annotations. These might include (1) tissues from disparate anatomical locations; and (2) spatial spots generated by the 10x platform. To accommodate these needs, ScopeViewer incorporates the widely used deep zoom format (a microsoft-maintained XML specification for viewing large images) and the advanced OpenSeadragon platform in DZI, SVS, and TIFF formats. ScopeViewer’s functionality extends beyond displaying multiple layers of large images at different magnification levels. It also supports dual views, a feature that enables side-by-side synchronized display (Supplementary Fig. 1). Additionally, ScopeViewer accommodates standard geometric annotations of various shapes, including lines, rectangles, ellipses, and polygons, through the integration of the Annotorious layer. These annotations can be manually created by human experts, such as pathologists, or generated automatically by AI software, like the HD-Yolo segmentation model [12]. The annotation data must be formatted in JSON, and users can conveniently specify the path to these layers within ScopeViewer’s online JSON editor, which provides instant feedback for any syntax errors. By seamlessly visualizing these diverse annotations, ScopeViewer facilitates biological spatial pattern exploration and hypothesis generation.

### Reduction of data transfer using a transpiled SQLite module

ST generates both high-resolution tissue image data and high-dimensional spatial molecular data, resulting in large datasets that are difficult to browse over the Internet. The process of transferring and processing such extensive ST data can be time-consuming and challenging. To overcome this obstacle, we incorporated a tailored SQLite database implementation that offers 2 key features: (1) a WebAssembly version of SQLite. This was transpiled from its original C codes and provides high execution speed within the browser, which significantly enhances performance [13, 14]. (2) It has the capacity to fetch expression quantities from the SQLite database through HTTP byte-range headers. This functionality minimizes data transfer from the SQLite virtual file system, making it more efficient. As a result of these optimizations, the webpage size is reduced from 180 M (original molecular data) to 17.2 M without cache, or 6.5 M with the cache. This inventive approach simplifies the visualization of ST data within browser-based applications, enhancing user experience and enabling more effective research analysis.

### Application: a breast cancer data from the 10X Visium platform

**Figure 1:**
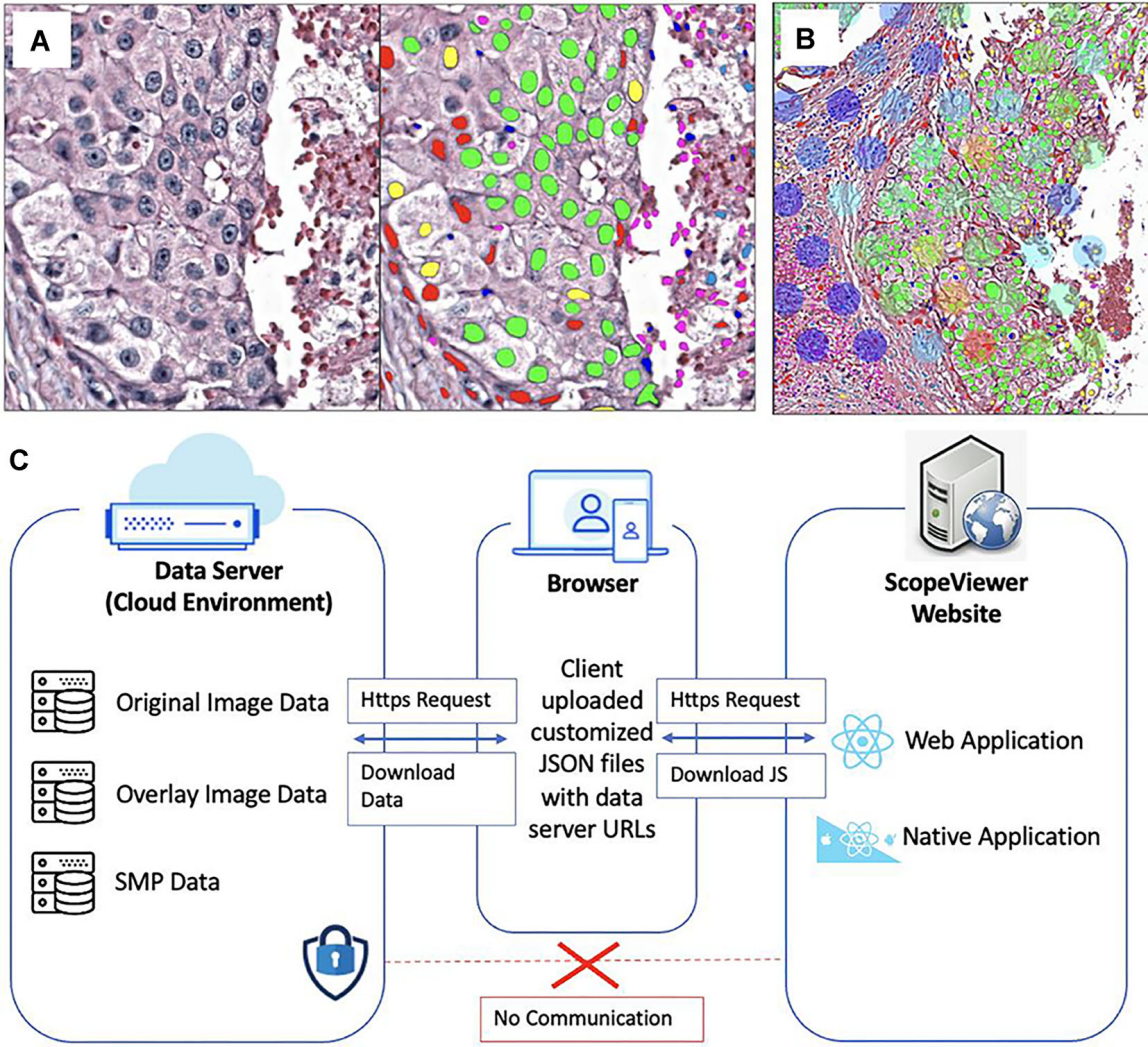
ScopeViewer for visualization ST data. (A) synchronized views for H&E pathology image and AI-facilitated cell segmentation; (B) efficiently overlaying gene expression features (here shown *FASN*, a breast cancer biomarker gene); (C) visualization will not leak genomic data to the ScopeViewer web server.

We demonstrate the use of ScopeViewer through a 10X Visium breast cancer FFPE sample [15]. To analyze the cell types and spatial distribution, we applied HD-Yolo, a deep-learning cell segmentation and classification algorithm on the whole slide image [12]. The image file measures 25,233 × 27,452 pixels and is 143 M in size. Both the original image and its annotations are stored in DZI formats and deployed as the default ScopeViewer Demo to showcase 3 key features: (1) the original H&E slides are displayed on the left, while the algorithm-annotated image appears on the right within a synchronized interface (Fig. 1A). At the highest magnification level, tumor nuclei, necrosis, red blood cells, and stroma cells are annotated in green, cyan, magenta, and red, respectively. (2) The molecular transcriptome data (the spots) can be overlaid on the image (Fig. 1B). This shows that the cancer biomarker gene FASN is expressed highly in the tumor region, clustering with tumor cells [16]. (3) No genomic data are transferred to the ScopeViewer web server, as data exchanges occur solely between the browser and the data server (Fig. 1C). This means the ScopeViewer web server instructs the user’s browser to retrieve and display relevant information, without accessing potentially sensitive data, as its website does not communicate with the data server.

### Application: a liver data from the 10X Visium platform

**Figure 2:**
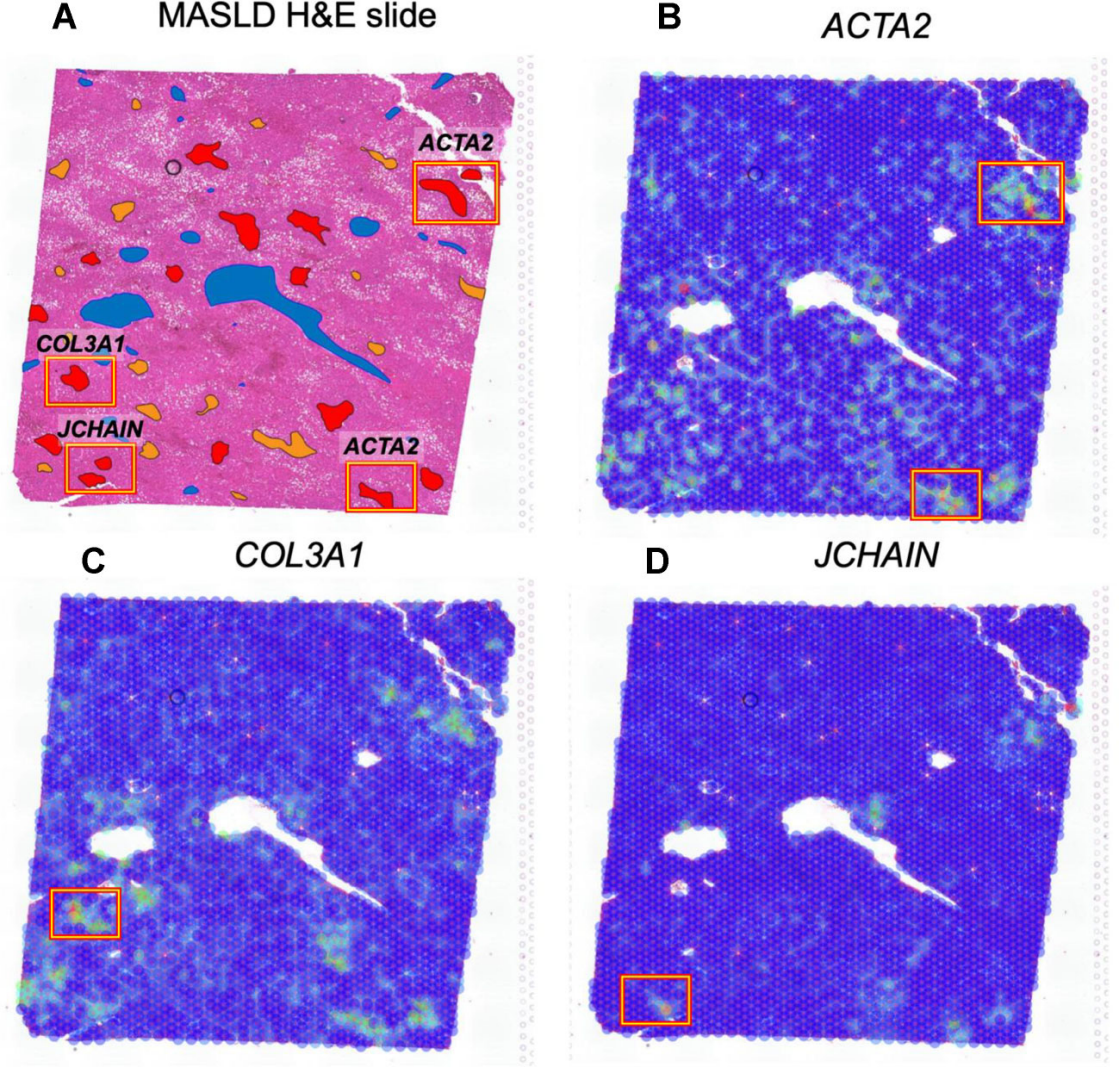
Heterogeneous pattern of spatial transcriptomics in MASLD. (A) Liver H&E slides with pathology annotations (blue: central vein; red: inflammatory/fibrotic portal tract; orange: non-inflammatory/fibrotic portal tract). Overexpression of activated myofibroblast marker genes, *ACTA2* (B) and *COL3A1* (C), and liver fibrogenesis signatures (D) overlap with inflammatory/fibrotic regions, demonstrating a heterogeneous biologic pattern. MASLD: metabolic dysfunction-associated steatotic liver disease.

To further demonstrate the versatility of ScopeViewer across different tissue types and biological contexts, we applied it to a liver Visium dataset, also accessible under the “Examples” tab on the ScopeViewer website. We fixed surgically resected clinical liver tissues in 10% formalin and embedded them in paraffin blocks. A 5-μm-thick tissue section was used for sequencing library preparation using the Visium FFPE Spatial Gene Expression kit (10x Genomics) and sequenced with the NextSeq 500 system (Illumina) to generate genome-wide transcriptome profile for each circular region called “spot” (55 μm in diameter) on the tissue section. Raw data were preprocessed using the Space Ranger software ver. 1.3.0 (10x Genomics) based on reference genome (hg38). The spots with >200 unique molecular identifier (UMI) counts were retained. Genes with a total UMI count <100 across all spots, expressed in <5 spots, and hemoglobin-related genes were excluded. We used ScopeViewer, which enables spatial visualization of pathogenic molecular dysregulations in stromal tissue in diseased liver. By using archived fixed surgical liver tissue affected with metabolic dysfunction-associated steatotic liver disease (MASLD), we performed Visium spatial transcriptome profiling. In MASLD liver, stromal tissue called the portal tract is the major site of chronic inflammation and fibrogenesis that drive disease progression toward organ failure and cancer development [17]. In 11 out of 17 portal tracts with deposition of more fibrous tissues and/or lymphocyte infiltration, overexpression of markers of activated myofibroblasts (*ACTA2* and *COL3A1*), the major driver of liver fibrogenesis [18], and/or plasma/B cells (*JCHAIN*), the major driver of pre-portal hepatic injury [19], are clearly visualized (Fig. 2). This example demonstrates utility of ScopeViewer as a convenient alternative to laborious and technically often challenging immuno-staining to visualize spatial heterogeneity in pathogenic molecular dysregulations from H&E image and spatial transcriptome profiling data.

## Conclusion and discussion

ScopeViewer is a general, feature-rich, cloud-based, and secure tool for visualizing large biological images, including ST datasets and H&E pathology slides. It specializes in secure, client-side rendering of large-scale ST data without the need to upload genomic information, a feature not commonly found in other tools. Compared to existing tools such as Vitessce [20], TissUUmaps [21], CZ Cellxgene [22], Cytomine [7], Napari [9], and Giotto [8], ScopeViewer offers a distinct combination of features: secure client-side rendering that avoids genomic data transfer to external servers, dual synchronized views optimized for pathology workflows, and browser-native SQLite support enabling efficient retrieval of large ST datasets without requiring server-side infrastructure. A detailed feature-by-feature comparison is provided in Supplementary Table 2. Furthermore, to our knowledge, ScopeViewer is among the first tools to support the browser native SQLite data format, enabling efficient retrieval of spatial data and flexible extension to general ST technology. This feature is enhanced by SQLite’s built-in R-tree spatial index. Lastly, ScopeViewer is optimized for user interaction and offers dual-view synchronized visualization for conveniently exploring raw images, histology, and molecular annotations, providing a novel enhancement for detailed tissue inspection. We envision that it will be a valuable resource for data exploration and sharing within the wider research community.

## Online resource

We used JavaScript and Node.js backend to implement the ScopeViewer. We provided the online resource source [10] and prepared Docker images at DockerHub [11]. Additionally, we provide examples that include single pathology images with hierarchical annotations, a list of pathology images, and ST data from the 10X Visium platform. ScopeViewer can also be customized to visualize new datasets for future digital pathology or ST studies. The codes are licensed under GNU General Public License v3.0

## Availability of source code and requirements

Project name: ScopeViewer

Project home page: https://cdc.biohpc.swmed.edu/scopeviewer

Operating system(s): Web browser

Programming language: JavaScript (Node.js version 16)

Other requirements: N/A

License: GNU General Public License v3.0

## Additional files

**Supplementary Figure S1:** ScopeViewer provides a convenient JSON editor for customized visualization. The ScopeViewer tool enables customizable visualization of spatial transcriptomics data via a JSON configuration file. We provide an editing feature supported by a JavaScript implementation based on the Monaco editor (under the MIT license), showcasing the following capabilities: (1) syntax colorization; (2) real-time syntax validation; (3) collapsible code blocks; (4) bracket matching for improved readability and error minimization.

**Supplementary Figure S2:** ScopeViewer allows visualization of annotations in a hierarchical structure. In tissue slides, multiple types of cells exist, and they can have a multiple-level hierarchical structure (e.g., cell lineages). ScopeViewer allows users to specify such tree structure in the JSON file. This illustration presents five distinct annotation layers (left) derived from the corresponding JSON source codes (right). Users have the flexibility to selectively display or conceal individual layers as required.

**Supplementary Figure S3:** User interface of ScopeViewer demonstrating rich visualization functions. The screenshots from ScopeViewer websites demonstrate useful features, including dual view of the same high-resolution H&E image, hierarchical annotations for tissue regions, gene-level transcriptome visualization, and a toolbar for annotation and bookmarking.

**Supplementary Table S1:** Comparison between SQLite and Zarr.

## List of abbreviations

H&E: hematoxylin and eosin; ST: spatial transcriptomics; MASLD: metabolic dysfunction-associated steatotic liver disease.

## Consent for publication

10X Visium breast cancer FFPE dataset is provided by 10X genomics which grants the researchers non-commercial access permission.

## Supplementary Material

giag074_Supplemental_File

giag074_Authors_Response_To_Reviewer_Comments_Original_Submission

giag074_GIGA-D-25-00402_Original_Submission

giag074_GIGA-D-25-00402_Revision_1

giag074_Reviewer_1_Report_Original_Submission Reviewer 1 -- 10/12/2025

giag074_Reviewer_1_Report_Revision_1 Reviewer 1 -- 5/22/2026

giag074_Reviewer_2_Report_Original_Submission Reviewer 2 -- 10/30/2025

giag074_Reviewer_3_Report_Original_Submission Reviewer 3 -- 11/3/2025

## Data Availability

Spatial transcriptome profiling dataset is publicly available: breast cancer dataset is from 10X Genomics public datasets, and liver disease dataset was generated in-house and has been deposited to the NCBI GEO (accession number: GSE278621). Additional technical details and demonstration of ScopeViewer functions are available in Supplementary texts and figures. To address the complexities of manual JSON configuration, we provide a Python helper script (database.py) that automates the creation of these JSON files, significantly lowering the barrier for users without prior coding or bioinformatics experience. Source codes to facilitate users to prepare ST data can be found at [23]. Comprehensive user guides on how to prepare data can also be found at [24]. Furthermore, a step-by-step video tutorial demonstrating this local setup and auto-configuration process is available in [25]. Alternatively, a Docker image of ScopeViewer is available online [10]. We also provide user support through GitHub issues to address researchers’ specific needs.
