# Supplementary material for: ScopeViewer: a browser-based solution for visualizing large biological images
Source: Gigascience. 2026 Jun 22;15:giag074. doi: 10.1093/gigascience/giag074 (PMC13369961; doi:10.1093/gigascience/giag074)
Supplement: giag074_Supplemental_File [file giag074_supplemental_file.docx]

**ScopeViewer: A Browser-Based Solution for Visualizing Large Biological Images**

Danni Luo^1^, Sophie Robertson^2^, Yuanchun Zhan^1^, Ruichen Rong^1^, Shidan Wang^1^, Xi Jiang^1^, Sen Yang^1^, Suzette Palmer^1^, Peiran Quan^1^, Hiroaki Kanzaki^3^, Yujin Hoshida^3^, Liwei Jia^4^, Qiwei Li^5^, Guanghua Xiao^1,*^, Xiaowei Zhan^1,*^

^1^ Quantitative Biomedical Research Center, Peter O’Donnell Jr. School of Public Health, UT Southwestern Medical Center

^2^ Paul Allen School of Computer Science & Engineering, University of Washington

^3^ Department of Internal Medicine, UT Southwestern Medical Center

^4^ Department of Pathology, UT Southwestern Medical Center

^5^ Department of Mathematics Sciences, University of Texas at Dallas.

*To whom correspondence should be addressed.

**Supplementary Materials**

**Supplementary Texts**

1. **A common file format that supports multiple spatial transcriptomics platforms**

**An overview of spatial transcriptomics platforms**

Cellular and molecular spatial organizations play essential roles in biological functions. The development of spatial transcriptomics (ST) techniques have achieved significant breakthroughs in recent years (Zhang, et al., 2021). These techniques enable high resolution transcriptome measurement with tissue spatial information, which provides new opportunities to advance our understanding of cellular and molecular spatial distributions (Crosetto, et al., 2015) and their relationships with diseases (Shah, et al., 2018). Popular ST techniques can be classified into two types: imaging-based and sequencing-based. Based on the single-molecule fluorescence *in situ* hybridization (FISH), the two most classical imaging-based ST techniques are sequential FISH (seqFISH) (Lubeck, et al., 2014) and multiplexed error-robust FISH (MERFISH) (Chen, et al., 2015). These techniques can measure the expression levels of hundreds to thousands of genes in individual cells. STARmap is a recently developed 3D intact-tissue RNA sequencing approach with single-cell resolution (Wang, et al., 2018). Sequencing-based techniques, such as spatial transcriptomics (Stahl, et al., 2016), 10x Visium platform, and high-definition spatial transcriptomics (HDST) (Vickovic, et al., 2019) use spatial barcode probes to capture RNA molecules and then synthesize and sequence their complementary DNA molecules. These techniques quantify the expression levels of the entire transcriptome by utilizing hundreds to thousands of barcode probes, referred to as spots, which collectively capture the profiles of cell. In summary, all the above technologies can quantify transcriptomics in a spatial context and we found that it is possible to store the measurements in a unified format for fast web-based visualizations.

**Data preparation**

ScopeViewer has the ability to generate spatial molecular profiling (SMP) layer, which overlays spot data on the histology image. To achieve this function, users need to provide corresponding SMP data for the image, which includes three essential files: 1) a gene expression matrix $Y$ with $P\times N$ dimensions, where $N$ denotes the number of spots or cells and $P$ denotes the number of genes. Each entry $y_{ij}$ is the read count for gene $i$ collected at spot/cell $j$; 2) a location table $T$ with $N$ rows and two columns. Each row contains the x and y coordinate of each spot/cell; and 3) a vector with length $P$ containing the list of gene names. All the data tables above need to be stored in “.csv” format. Users need to use the provided Python script to convert all files into three tables (‘smp_count_sql’, ‘smp_loc_sql’, and ‘gene_list_sql’) in a SQlite database file, and then provide the path of generated .db file with "smp_layer" key in a .json file. Detailed instructions and example files can be found on our website.

1. **Transpile SQLite as a web module**

Spatial transcriptomics datasets often range from megabytes to gigabytes in size. While this scale of disk space is easily manageable for desktop-based visualization software, it poses a significant challenge for cloud-based applications like ScopeViewer due to the slow transfer of large data volumes from the internet to the user's browser.

To overcome this obstacle, we adopted the use of the SQLite database, transpiled to WebAssembly (WASM) for efficient binary execution and enhanced computational speed. We further leveraged an open-source implementation to selectively retrieve B-tree blocks from the underlying SQLite database file, thereby transferring only necessary blocks using the byte range feature commonly found in web servers or cloud storage services (e.g., Amazon S3).

This process results in transferring only a small fraction of transcriptomics data visible to the user over the internet. In our benchmarks, we typically observed a reduction in data transfer from 1/10th to 1/100th of the original data, demonstrating that our transpiled SQLite approach substantially decreases the bandwidth required for visualizing spatial transcriptomics datasets.

1. **Comparing Spatial Data Formats**

Transpiled SQLite and Zarr serve different but complementary roles in spatial data storage and access. With HTTP range requests, SQLite can now support efficient random retrieval, making it strong for tabular or interval-based genomics such as variants, gene annotations, and metadata, especially since it natively supports SQL queries and R-tree indexing. However, SQLite is less suited for very large multidimensional data like coverage tracks or single-cell matrices, where Zarr excels. Zarr’s chunked, compressed array structure is cloud-native and enables scalable, parallel access to large numerical datasets, but it lacks native SQL querying and R-tree spatial indexing. In this manuscript, we unique implemented transpile SQLite with the support of HTTP range request. We demonstrated that it can efficiently retrieve metadata and support spatial queries using the SQL syntax.

**Supplementary Table 1: comparison between SQLite and Zarr.**

| Feature / Use Case | SQLite (transpiled + HTTP) | Zarr |
| --- | --- | --- |
| Data model | Relational tables (rows/columns) | Chunked N-dimensional arrays |
| Query language | SQL (joins, filters, aggregates) | None (array slicing only) |
| Spatial indexing | R-tree built-in | Not native (only chunk partitioning) |
| Random access | Via HTTP range requests | Native via chunked access |

**Supplementary Figure 1. ScopeViewer provides a convenient JSON editor for customized visualization.** The ScopeViewer tool enables customizable visualization of spatial transcriptomics data via a JSON configuration file. We provide an editing feature supported by a JavaScript implementation based on the Monaco editor (under the MIT license), showcasing the following capabilities: (1) Syntax colorization; (2) Real-time syntax validation; (3) Collapsible code blocks; (4) Bracket matching for improved readability and error minimization.

**
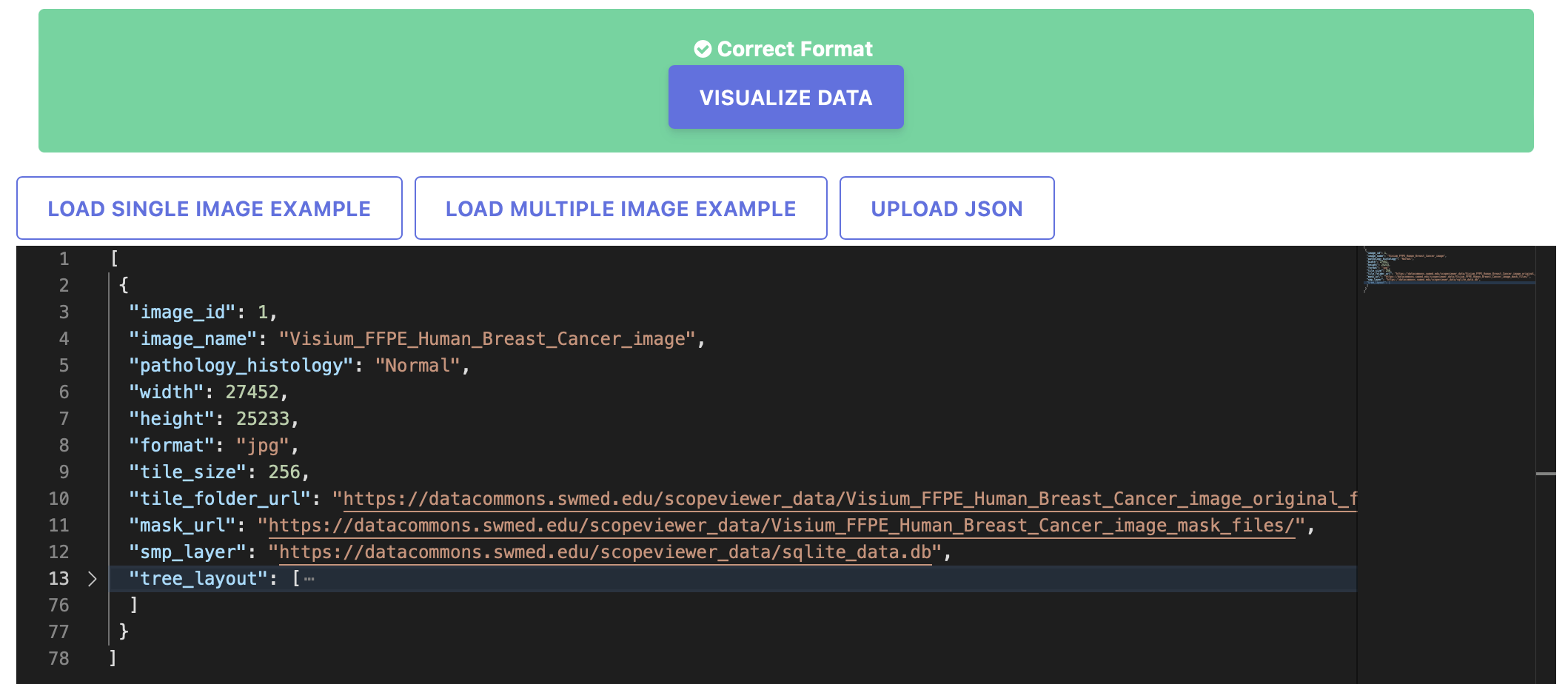
**

**Supplementary Figure 2. ScopeViewer allows visualization of annotations in a**

**hierarchical structure.** In tissue slides, multiple types of cells exist, and they can have a multiple level hierarchical structure (e.g., cell lineages). ScopeViewer allow users to specify such tree-structure in the JSON file. This illustration presents five distinct annotation layers (left) derived from the corresponding JSON source codes (right). Users have the flexibility to selectively display or conceal individual layers as required.

**
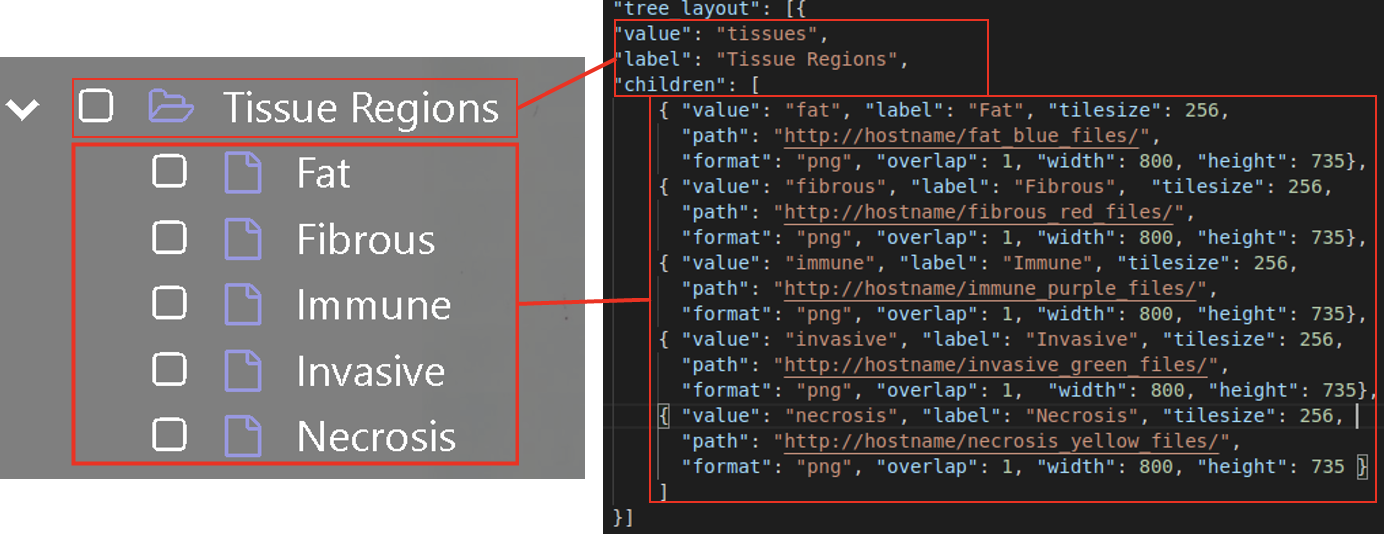
**

**Supplementary Figure 3. User interface of ScopeViewer demonstrating rich visualization functions.** The screenshot from ScopeViewer websites demonstrate useful features including: dual view of the same high-resolution H&E image, hierarchical annotations for tissue regions, gene-level transcriptome visualization, and a toolbar for annotation and bookmarking.

**
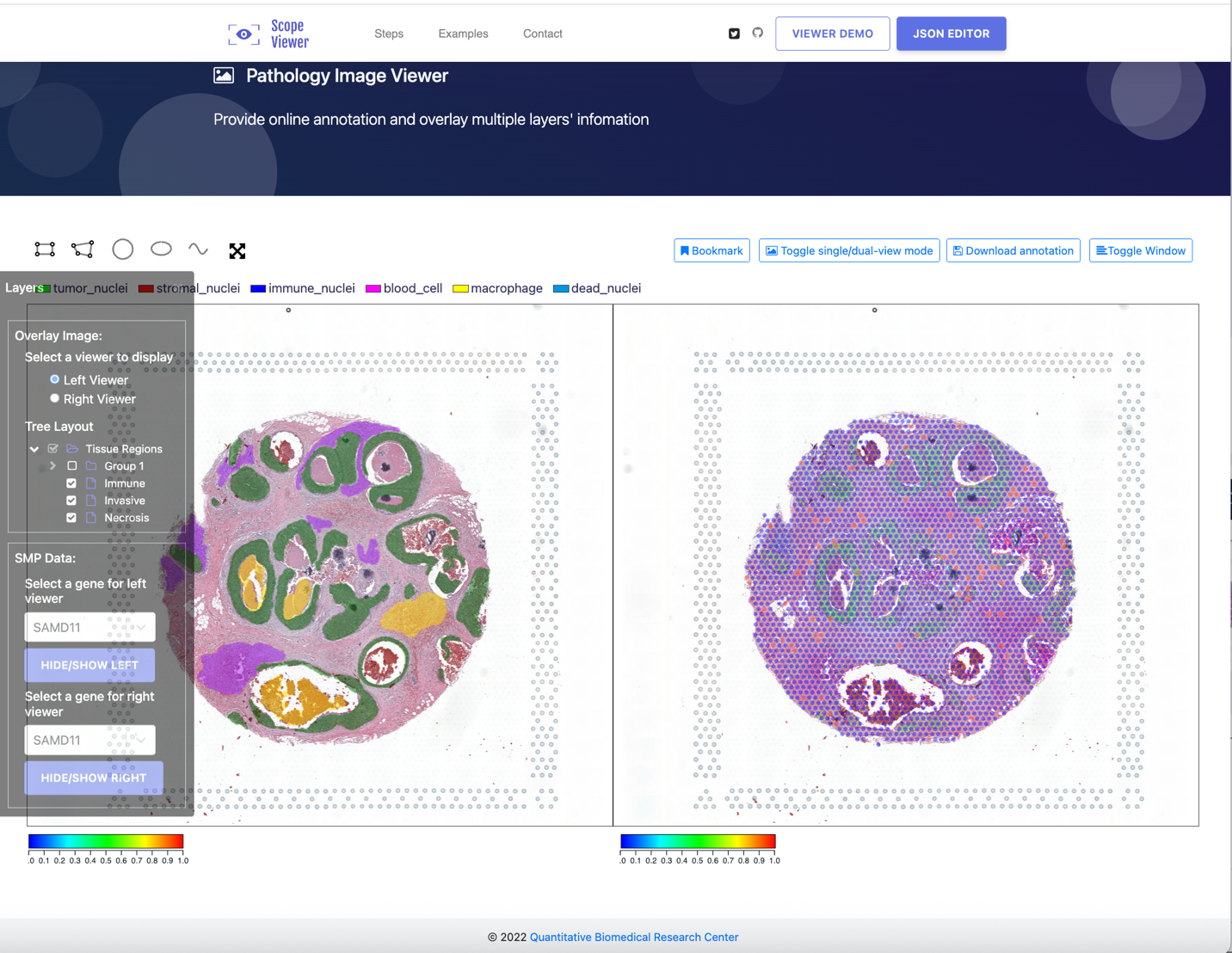
**

**Reference**

Chen, K.H.*, et al.* RNA imaging. Spatially resolved, highly multiplexed RNA profiling in single cells. *Science* 2015;348(6233):aaa6090.

Crosetto, N., Bienko, M. and van Oudenaarden, A. Spatially resolved transcriptomics and beyond. *Nat Rev Genet* 2015;16(1):57-66.

Lubeck, E.*, et al.* Single-cell in situ RNA profiling by sequential hybridization. *Nature methods* 2014;11(4):360-361.

Shah, S.*, et al.* Dynamics and Spatial Genomics of the Nascent Transcriptome by Intron seqFISH. *Cell* 2018;174(2):363-376 e316.

Stahl, P.L.*, et al.* Visualization and analysis of gene expression in tissue sections by spatial transcriptomics. *Science* 2016;353(6294):78-82.

Vickovic, S.*, et al.* High-definition spatial transcriptomics for in situ tissue profiling. *Nature methods* 2019;16(10):987-990.

Wang, X.*, et al.* Three-dimensional intact-tissue sequencing of single-cell transcriptional states. *Science* 2018;361(6400).

Zhang, M.*, et al.* Spatial molecular profiling: platforms, applications and analysis tools. *Brief Bioinform* 2021;22(3).
