# Supplementary material for: ScopeViewer: a browser-based solution for visualizing large biological images
Source: Gigascience. 2026 Jun 22;15:giag074. doi: 10.1093/gigascience/giag074 (PMC13369961; doi:10.1093/gigascience/giag074)

GigaScience

ScopeViewer: A Browser-Based Solution for Visualizing Large Biological Images

--Manuscript Draft--

**Manuscript Number:**	GIGA-D-25-00402
**Full Title:**	ScopeViewer: A Browser-Based Solution for Visualizing Large Biological Images
**Article Type:**	Research

**Funding Information:**		
	National Institute of Nursing Research (R01GM140012)	Dr. Guanghua Xiao
	National Institutes of Health (R01GM141519)	Dr. Guanghua Xiao
	National Institutes of Health (R01DE030656)	Dr. Guanghua Xiao
	National Institutes of Health (U01CA249245)	Dr. Guanghua Xiao
	National Institutes of Health (U01AI169298)	Dr. Xiaowei Zhan

Abstract:

Background

Spatial transcriptomics (ST) enables a high-resolution interrogation of molecular characteristics within specific spatial contexts and tissue morphology. Despite its potential, visualization of ST data is a challenging task due to the complexities in handling, sharing, and visualizing large image datasets together with molecular information.

Results

We introduce ScopeViewer, a browser-based software designed to overcome these challenges. ScopeViewer offers the following functionalities: (1) It visualizes large image data and associated annotations at various zoom levels, allowing for intricate exploration of the data; (2) It enables dual interactive viewing of the original images along with their annotations, providing a comprehensive understanding of the context; (3) It displays spatial molecular features with optimized bandwidth, ensuring a smooth user experience; and (4) It bolsters data security by circumventing data transfers.

Conclusions and Discussions

ScopeViewer offers the research community a convenient, powerful, and secure software for high-resolution images including pathology images and spatial transcriptomics. It serves as an open-source platform for imaging-based research. Future enhancements and new features will be shared on GitHub by the creators and are open for contributions from other researchers.

Availability and Implementation

ScopeViewer is freely available on the web at: https://cdc.biohpc.swmed.edu/scopeviewer . We offer detailed documentation that guides users through preparing and specifying data locations in an online JSON editor. Additionally, we provide example configurations for users to reference. Online demos are available to help users get started at https://cdc.biohpc.swmed.edu/scopeviewer/imageviewer. ScopeViewer can be used as a lightweight browsing application without requiring users to set up software dependencies.

**Corresponding Author:**	Xiaowei Zhan<br>University of Texas Southwestern Medical School<br>Dallas, TX UNITED STATES
**Corresponding Author Secondary Information:**	
**Corresponding Author's Institution:**	University of Texas Southwestern Medical School

Powered by Editorial Manager® and ProduXion Manager® from Aries Systems Corporation

Corresponding Author's Secondary Institution:	
First Author:	Xiaowei Zhan
First Author Secondary Information:	
Order of Authors:	Xiaowei Zhan
	Danni Luo
	Yuanchun Zhan
	Sophie Robertson
	Ruichen Rong
	Shidan Wang
	Xi Jiang
	Sen Yang
	Suzette Palmer
	Liwei Jia
	Qiwei Li
	Guanghua Xiao
Order of Authors Secondary Information:	
Additional Information:	
Question	Response
Are you submitting this manuscript to a special series or article collection?	No
**Experimental design and statistics**<br><br>Full details of the experimental design and statistical methods used should be given in the Methods section, as detailed in our Minimum Standards Reporting Checklist. Information essential to interpreting the data presented should be made available in the figure legends.<br><br>Have you included all the information requested in your manuscript?	Yes
**Resources**<br><br>A description of all resources used, including antibodies, cell lines, animals and software tools, with enough information to allow them to be uniquely identified, should be included in the Methods section. Authors are strongly	Yes

Powered by Editorial Manager® and ProduXion Manager® from Aries Systems Corporation

encouraged to cite Research Resource Identifiers (RRIDs) for antibodies, model organisms and tools, where possible.<br><br>Have you included the information requested as detailed in our Minimum Standards Reporting Checklist?	
**Availability of data and materials**<br><br>All datasets and code on which the conclusions of the paper rely must be either included in your submission or deposited in publicly available repositories (where available and ethically appropriate), referencing such data using a unique identifier in the references and in the “Availability of Data and Materials” section of your manuscript.<br><br>Have you have met the above requirement as detailed in our Minimum Standards Reporting Checklist?	Yes
GigaScience has policies and guidelines in place for the use of generative AI-writing tools such as ChatGPT. If you have used such writing tools to assist with writing the manuscript this must be declared and cited in the text. Authors should not list AI-writing tools and other AI-assisted technologies as an author or co-author and should acknowledge that they are fully responsible for text generated or refined by AI-writing tools.<p><br><br>A summary of use (particularly in the introduction or among methods) needs to be included at the end of the paper, and the outputs should also be included as a supplementary file hosted in GigaDB or other open repositories. Please read our guidelines for more information. <p><br><br>By submitting to GigaScience, you are aware of the journal's AI-writing tools policy, and if you have declared use of such tools below, you have acknowledged this where appropriate in your manuscript and have made a summary of use and	No

Powered by Editorial Manager® and ProduXion Manager® from Aries Systems Corporation

outputs available. <p><br><b>AI-assisted writing tools have been used in the preparation of this manuscript?	

Powered by Editorial Manager® and ProduXion Manager® from Aries Systems Corporation

Manuscript

Click here to access/download;Manuscript;manuscript clean 0820.docx

ScopeViewer: A Browser-Based Solution for Visualizing Large Biological Images

Danni Luo[1], Sophie Robertson[2], Yuanchun Zhan[1], Ruichen Rong[1], Shidan Wang[1], Xi Jiang[1], Sen Yang[1], Suzette Palmer[1], Liwei Jia[3], Qiwei Li[4], Guanghua Xiao[1,*], Xiaowei Zhan[1,*]

[1] Quantitative Biomedical Research Center, Peter O'Donnell Jr. School of Public Health, UT Southwestern Medical Center

[2] Paul Allen School of Computer Science & Engineering, University of Washington

[3] Department of Pathology, UT Southwestern Medical Center

[4] Department of Mathematics Sciences, University of Texas at Dallas.

*To whom correspondence should be addressed.

Abstract

Background

Spatial transcriptomics (ST) enables a high-resolution interrogation of molecular characteristics within specific spatial contexts and tissue morphology. Despite its potential, visualization of ST data is a challenging task due to the complexities in handling, sharing, and visualizing large image datasets together with molecular information.

Results

We introduce ScopeViewer, a browser-based software designed to overcome these challenges. ScopeViewer offers the following functionalities: (1) It visualizes large image data and associated annotations at various zoom levels, allowing for intricate exploration of the data; (2) It enables dual interactive viewing of the original images along with their annotations, providing a comprehensive understanding of the context; (3) It displays spatial molecular features with optimized bandwidth, ensuring a smooth user experience; and (4) It bolsters data security by circumventing data transfers.

Conclusions and Discussions

ScopeViewer offers the research community a convenient, powerful, and secure software for high-resolution images including pathology images and spatial transcriptomics. It serves as an open-source platform for imaging-based research. Future enhancements and new features will be shared on GitHub by the creators and are open for contributions from other researchers.

Availability and Implementation

ScopeViewer is freely available on the web at: https://cdc.biohpc.swmed.edu/scopeviewer . We offer detailed documentation that guides users through preparing and specifying data locations in an online JSON editor. Additionally, we provide example configurations for users to reference. Online demos are available to help users get started at https://cdc.biohpc.swmed.edu/scopeviewer/imageviewer. ScopeViewer can be used as a lightweight browsing application without requiring users to set up software dependencies.

Contact

Xiaowei.Zhan@UTSouthwestern.edu, Guanghua.Xiao@UTSouthwestern.edu

1 Introduction

Spatial transcriptomics (ST) technologies have made significant advancements in recent years [1]. ST techniques offer high-resolution transcriptome measurements with spatial information within tissues, thereby opening new avenues for understanding cellular and molecular spatial distributions [2], and their associated links to diseases [3]. A typical ST dataset pairs with high-resolution images (e.g., H&E pathology slides), often comprising millions of pixels. This facilitates a dual visualization of cellular and tissue structures alongside quantitative molecular features, including gene expression and protein abundance. Examining molecular characteristics within spatial and morphological contexts could pave the way for new biological discoveries. A comprehensive tool for visualizing ST data will streamline data exploration and analysis, aiding researchers in comprehending molecular features within specific biological contexts.

Working with high-resolution tissue images and ST data introduces significant challenges due to the huge volume of these datasets. Consider a standard pathology image of 20,000 by 20,000 pixels, 0.5 microns per pixel, which can amass a file size of approximately one gigabyte. This large size complicates both the image's transfer and visualization, often requiring specialized software tools. Moreover, there is a high degree of complexity inherent in visualizing high-dimensional molecular features alongside the intricate cell and tissue structures. As a result, many software packages [4-6] currently require specific preprocessing steps to display molecular details alongside high-resolution tissue images simultaneously. Further complications arise from software interfaces that require tedious manual input from users to toggle the visibility of data layers. A more streamlined solution might include offering a dual-view approach. This would allow users to see the data layer in one view, while simultaneously hiding it in another, with synchronized panning and zooming capabilities. Lastly, the ability for researchers to explore ST data on their own systems, without uploading or sharing data externally, is an important consideration. This not only bolsters data security but also enhances user accessibility. To address these prevalent challenges, we developed ScopeViewer, a browser-based visualization software, available at: https://cdc.biohpc.swmed.edu/scopeviewer. A docker image is also freely available at: https://hub.docker.com/r/utsw1qbrc/scopeviewer.

2 Methods and Results

ScopeViewer operates as a web application, requiring nothing more than a web browser for its execution. It was designed using ReactJS JavaScript framework. To utilize ScopeViewer, users simply navigate to the website and input the image information and

ST data from a local path, using the JSON syntax. ScopeViewer generates an interactive user interface directly within the web browser. The platform's design leverages the versatility of web browsers, thereby eliminating the need for users to install specific software on their hardware.

2.1 Supports for multiple imaging formats

When conducting pathology image analysis or exploring ST data, it is crucial to view the image at varying magnification levels. Additionally, users often need to overlay various sources for annotations. These might include (1) tissues from disparate anatomical locations; and (2) spatial spots generated by the 10x platform. To accommodate these needs, ScopeViewer incorporates the widely used deep zoom format (a Microsoft-maintained XML specification for viewing large images) and the advanced OpenSeaDragon platform [7] in DZI, SVS, and TIFF formats. ScopeViewer's functionality extends beyond displaying multiple layers of large images at different magnification levels. It also supports dual views, a feature that enables side-by-side synchronized display (Supplementary Fig. 1). Additionally, ScopeViewer accommodates user annotations of various shapes, including lines, rectangles, ellipses, and polygons. These annotations must be included in JSON and can be added using the Annotorious layer. Users can conveniently specify the path to these layers within an online JSON editor in ScopeViewer, which provides instant feedback for any syntax errors. This feature enhances user-friendliness and ensures accurate data input.

2.2 Reduction of data transfer using a transpiled SQLite module

ST generates both high-resolution tissue image data and high-dimensional spatial molecular data, resulting in large datasets that are difficult to browse over the Internet.

The process of transferring and processing such extensive ST data can be time-consuming and challenging. To overcome this obstacle, we incorporated a tailored SQLite database implementation that offers two key features: (1) a WebAssembly version of SQLite. This was transpiled from its original C codes and provides high execution speed within the browser, which significantly enhances performance [8, 9]. (2) It has the capacity to fetch expression quantities from the SQLite database through HTTP Byte-Range headers. This functionality minimizes data transfer from the SQLite Virtual File System (VFS), making it more efficient. As a result of these optimizations, the webpage size is reduced from 180M (original molecular data) to 17.2M without cache, or 6.5M with the cache. This inventive approach simplifies the visualization of ST data within browser-based applications, enhancing user experience and enabling more effective research analysis.

2.3 Application: a breast cancer data from the 10X Visium platform

We demonstrate the use of ScopeViewer through a 10X Visium breast cancer FFPE sample [10]. To analyze the cell types and spatial distribution, we applied HD-Yolo, a deep-learning cell segmentation and classification algorithm on the whole slide image [11]. The image file measures 25,233 x 27,452 pixels and is 143M in size. Both the original image and its annotations are stored in DZI formats and deployed as the default ScopeViewer Demo to showcase three key features: (1) The original H&E slides are displayed on the left, while the algorithm-annotated image appears on the right within a synchronized interface (Fig. 1A). At the highest magnification level, tumor nuclei, necrosis, red blood cells, and stroma cells are annotated in green, cyan, magenta, and red, respectively. (2) The molecular transcriptome data (the spots) can be overlaid on the

image (Fig. 1B). This shows that the cancer biomarker gene FASN is expressed highly in the tumor region, clustering with tumor cells [12]. (3) No genomic data are transferred to the ScopeViewer web server, as data exchanges occur solely between the browser and the data server (Fig. 1C). This means the ScopeViewer web server instructs the user's browser to retrieve and display relevant information, without accessing potentially sensitive data, as its website does not communicate with the data server.

3 Conclusion

ScopeViewer is a general, feature-rich, cloud-based, and secure tool for visualizing large biological images including ST datasets and H&E pathology slides. It specializes in secure, client-side rendering of large-scale spatial transcriptomics data without the need to upload genomic information, a feature not commonly found in other tools. Furthermore, ScopeViewer is the only tool that supports the browser native SQLite data format, enabling efficient retrieval of spatial data. This feature is enhanced by SQLite's built-in R-tree spatial index. Lastly, ScopeViewer is optimized for user interaction and offers dual-view synchronized visualization for comparing raw images and annotations, providing a novel enhancement for detailed tissue inspection. We envision that it will be a valuable resource for data exploration and sharing within the wider research community.

Online resource

We used Javascript and nodejs backend to implement the ScopeViewer. We provided the online resource source at https://cdc.biohpc.swmed.edu/scopeviewer and prepared Docker images at DockerHub https://hub.docker.com/r/utsw1qbrc/scopeviewer.

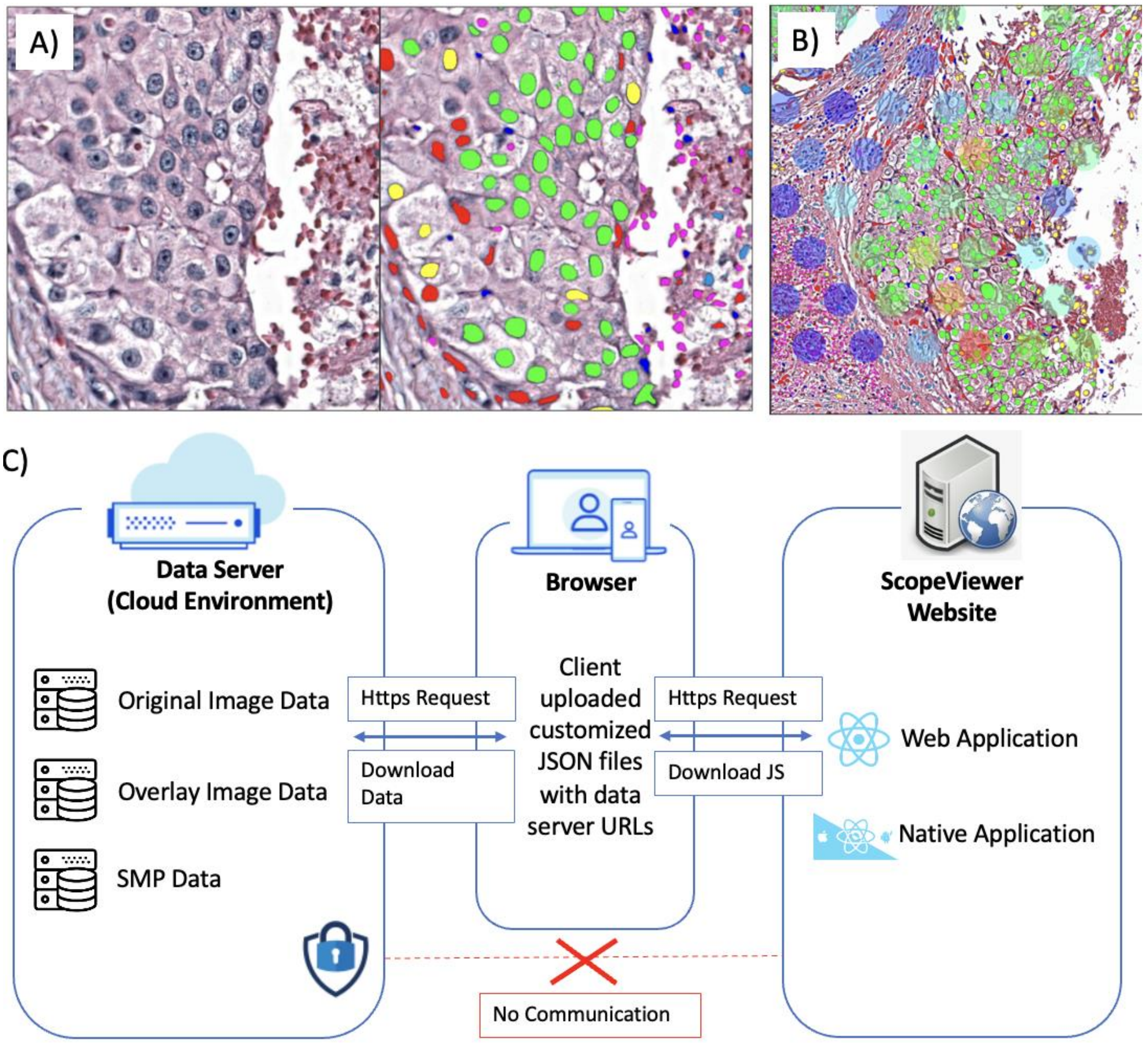

Figure 1: ScopeViewer for visualization ST data.
(1) synchronized views for H&E pathology image and AI facilitated cell segmentation; (2) efficiently overlaying gene expression features (here shown *FASN*, a breast cancer biomarker gene); (3) visualization will not leak genomic data to the ScopeViewer webserver.

Additionally, we provide examples that include single pathology images with hierarchical annotations, a list of pathology images, and ST data from the 10X Visium platform. ScopeViewer can also be customized to visualize new datasets for future digital

pathology or ST studies. The codes are licensed under GNU General Public License v3.0.

Availability of source code and requirements

Project name: ScopeViewer
Project home page: https://cdc.biohpc.swmed.edu/scopeviewer
Operating system(s): Web browser
Programming language: Javascript (nodejs version 16)
Other requirements: N/A
License: GNU General Public License v3.0

Availability of supporting data and materials

Additional technical details and demonstration of ScopeViewer functions are available in Supplementary Texts and Figures. Source codes to facilitate users to prepare ST data can be found at: https://cdc.biohpc.swmed.edu/scopeviewer/database.py. User guides on how to prepare data can be found at: https://cdc.biohpc.swmed.edu/scopeviewer/prepareData.

List of abbreviations

H&E: Hematoxylin and Eosin
ST: Spatial transcriptomics

Ethic

Not applicable.

Consent for publication

10X Visium breast cancer FFPE dataset is provided by 10X genomics which grants the researchers non-commercial access permission.

Competing Interests

No competing interest was reported by any author.

Funding

This study is partially supported by the National Institutes of Health (U01AI169298 [XZ], R01HG011035 [XZ], R01GM140012 [GX], R01GM115473 [GX], R01DE030656 [GX], 1U01CA249245 [GX]) and the Cancer Prevention and Research Institute of Texas (CPRIT RP230330 [GX]).

Author's Contributions

D.L., S.R., and Y. Z. performed the experiment and wrote software. R.R., S, W., S. Y., S. P., L. J., and Q.L. provided resources and helpful discussions. D.L., G.X. and X.Z. designed the experiment, performed data analysis, and wrote the manuscript.

Acknowledgments

We thank Ismael Villanueva-Miranda, Jonathan Wang, Fangjiang Wu, Shengjie Yang, and Qinbo Zhou for their contribution to the software development and/or comments on the manuscript.

Reference

1. Zhang M, Sheffield T, Zhan X, Li Q, Yang DM, Wang Y, et al. Spatial molecular profiling: platforms, applications and analysis tools. Brief Bioinform. 2021;22 3 doi:10.1093/bib/bbaa145.
2. Crosetto N, Bienko M and van Oudenaarden A. Spatially resolved transcriptomics and beyond. Nat Rev Genet. 2015;16 1:57-66. doi:10.1038/nrg3832.
3. Shah S, Takei Y, Zhou W, Lubeck E, Yun J, Eng CL, et al. Dynamics and Spatial Genomics of the Nascent Transcriptome by Intron seqFISH. Cell. 2018;174 2:363-76 e16. doi:10.1016/j.cell.2018.05.035.
4. Maree R, Rollus L, Stevens B, Hoyoux R, Louppe G, Vandaele R, et al. Collaborative analysis of multi-gigapixel imaging data using Cytomine. Bioinformatics. 2016;32 9:1395-401. doi:10.1093/bioinformatics/btw013.

5. Dries R, Zhu Q, Dong R, Eng CL, Li H, Liu K, et al. Giotto: a toolbox for integrative analysis and visualization of spatial expression data. Genome biology. 2021;22 1:78. doi:10.1186/s13059-021-02286-2.
6. Chiu C-L and Clack N. Napari: a Python multi-dimensional image viewer platform for the research community. Microscopy and Microanalysis. 2022;28 S1:1576-7.
7. OpenSeaDragon: OpenSeadragon. https://openseadragon.github.io (2023).
8. Andrés BF and Pérez M. Transpiler-based architecture for multi-platform web applications. In: *2017 IEEE Second Ecuador Technical Chapters Meeting (ETCM)* 16-20 Oct. 2017 2017, pp.1-6.
9. Rossberg A. Webassembly specification. WebAssembly Community Group. 2021.
10. Janesick A, Shelansky R, Gottscho AD, Wagner F, Rouault M, Beliakoff G, et al. High resolution mapping of the breast cancer tumor microenvironment using integrated single cell, spatial and in situ analysis of FFPE tissue. bioRxiv. 2022:2022.10.06.510405. doi:10.1101/2022.10.06.510405.
11. Rong R, Sheng H, Jin KW, Wu F, Luo D, Wen Z, et al. A Deep Learning Approach for Histology-Based Nucleus Segmentation and Tumor Microenvironment Characterization. Mod Pathol. 2023;36 8:100196. doi:10.1016/j.modpat.2023.100196.
12. He B, Bergenstrahle L, Stenbeck L, Abid A, Andersson A, Borg A, et al. Integrating spatial gene expression and breast tumour morphology via deep learning. Nat Biomed Eng. 2020;4 8:827-34. doi:10.1038/s41551-020-0578-x.

Click here to access/download
Supplementary Material
Supplementary Materials final.docx

response to previous reviewer

Click here to access/download
Supplementary Material
Response to reviewers 0923.docx

Cover letter Click here to access/download;Personal Cover;CoverLetter.docx

UT SOUTHWESTERN MEDICAL CENTER

Editorial Board

September 23, 2025

GigaScience

Dear Editor:

We are submitting a revised manuscript"ScopeViewer: A Browser-Based Solution for Visualizing Large Biological Images" by Luo *et al*., which we respectfully ask that you consider for publication in *GigaScience*. This submission is an extensive revision following previous communication regarding manuscript ID GIGA-D-23-00318.The manuscript presents original research that has not been published or presented publicly before.

Recent advancements in technologies like digital pathology and spatial transcriptomics have led to the rapid emergence of large biological images. These technologies enable detailed examination of fine-scale characteristics within specific spatial contexts, generating data sets paired with high-resolution images, often with millions of pixels. However, visualizing these complex data sets alongside molecular information poses significant challenges in terms of management, sharing, and visualization.

To tackle these challenges, we present ScopeViewer, a browser-based tool designed for efficient handling and visualization of large image data sets. It enables exploration with variable zoom levels, supports dual interactive viewing of original images and annotations, providing a comprehensive understanding of the data context.

Furthermore, ScopeViewer enhances the user experience by optimizing the display of spatial molecular features, ensuring a smooth and intuitive interaction. Significantly, it also heightens data security by circumventing the need for data transfers, effectively reducing potential risks. The potential of ScopeViewer was demonstrated using a 10X Visium breast cancer FFPE sample. Through this, we showcased key features including synchronized viewing of the original and algorithm-annotated images, overlay of molecular transcriptome data on images, and secure data handling where exchanges occur solely between the user's browser and the data server.

As a cloud-based tool, ScopeViewer is not just rich in features but also provides an efficient and secure platform for visualizing large images. Given these capabilities, we envision that ScopeViewer will serve as a valuable resource for data exploration and sharing within the broader research community, facilitating further advancements in the study and understanding of spatial transcriptomics.

We respectfully suggest that the following individuals have the expertise to fairly review our manuscript:

Dr. Mingyao Li, Professor, Department of Biostatistics, Epidemiology and Informatics, University of Pennsylvania mingyao@pennmedicine.upenn.edu *Reason: Leading expert in spatial transcriptomics data analysis*

Dr. Xiang Zhou, Associate Professor, Department of Biostatistics, University of Michigan xzhousph@umich.edu *Reason: Leading expert in spatial transcriptomics data analysis*

All co-authors have reviewed the submitted manuscript and have indicated their approval of the findings and conclusions. Please feel free to contact us if you need additional information.

Thank you for your consideration of our work.

Sincerely,

Xiaowei Zhan

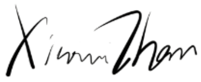

Supplement: giag074_GIGA-D-25-00402_Original_Submission [file giag074_giga-d-25-00402_original_submission.pdf]
