# Supplementary material for: ScopeViewer: a browser-based solution for visualizing large biological images
Source: Gigascience. 2026 Jun 22;15:giag074. doi: 10.1093/gigascience/giag074 (PMC13369961; doi:10.1093/gigascience/giag074)
Supplement: giag074_GIGA-D-25-00402_Revision_1 [file giag074_giga-d-25-00402_revision_1.pdf]

GigaScience

ScopeViewer: A Browser-Based Solution for Visualizing Large Biological Images

--Manuscript Draft--

**Manuscript Number:**	GIGA-D-25-00402R1
**Full Title:**	ScopeViewer: A Browser-Based Solution for Visualizing Large Biological Images
**Article Type:**	Research
**Funding Information:**	National Institute of Nursing Research (R01GM140012) — Dr. Guanghua Xiao<br>National Institutes of Health (R01GM141519) — Dr. Guanghua Xiao<br>National Institutes of Health (R01DE030656) — Dr. Guanghua Xiao<br>National Institutes of Health (U01CA249245) — Dr. Guanghua Xiao<br>National Institutes of Health (U01AI169298) — Dr. Xiaowei Zhan

Abstract:

Background

Spatial transcriptomics (ST) enables a high-resolution interrogation of molecular characteristics within specific spatial contexts and tissue morphology. Despite its potential, visualization of ST data is a challenging task due to the complexities in handling, sharing, and visualizing large image datasets together with molecular information.

Results

We introduce ScopeViewer, a browser-based software designed to overcome these challenges. ScopeViewer offers the following functionalities: (1) It visualizes large image data and associated annotations at various zoom levels, allowing for intricate exploration of the data; (2) It enables dual interactive viewing of the original images along with their annotations, providing a comprehensive understanding of the context; (3) It displays spatial molecular features with optimized bandwidth, ensuring a smooth user experience; and (4) It bolsters data security by circumventing data transfers.

Conclusions and Discussions

ScopeViewer offers the research community a convenient, powerful, and secure software for high-resolution images including pathology images and spatial transcriptomics. It serves as an open-source platform for imaging-based research. Future enhancements and new features will be shared on GitHub by the creators and are open for contributions from other researchers.

Availability and Implementation

ScopeViewer is freely available on the web at: https://cdc.biohpc.swmed.edu/scopeviewer . We offer detailed documentation that guides users through preparing and specifying data locations in an online JSON editor. Additionally, we provide example configurations for users to reference. Online demos are available to help users get started at https://cdc.biohpc.swmed.edu/scopeviewer/imageviewer. ScopeViewer can be used as a lightweight browsing application without requiring users to set up software dependencies.

**Corresponding Author:**	Xiaowei Zhan<br>University of Texas Southwestern Medical School<br>Dallas, TX UNITED STATES
**Corresponding Author Secondary Information:**	
**Corresponding Author's Institution:**	University of Texas Southwestern Medical School

Powered by Editorial Manager® and ProduXion Manager® from Aries Systems Corporation

Corresponding Author's Secondary Institution:	
First Author:	Xiaowei Zhan
First Author Secondary Information:	
Order of Authors:	Xiaowei Zhan
	Danni Luo
	Yuanchun Zhan
	Sophie Robertson
	Ruichen Rong
	Shidan Wang
	Xi Jiang
	Sen Yang
	Suzette Palmer
	Peiran Quan
	Hiroaki Kanzaki
	Yujin Hoshida
	Liwei Jia
	Qiwei Li
	Guanghua Xiao
Order of Authors Secondary Information:	
Response to Reviewers:	Dear reviewers,<br><br>Please find the uploaded "Response to reviewer" for a well-formatted response. Thank you for your constructive comments.
Additional Information:	
Question	Response
Are you submitting this manuscript to a special series or article collection?	No
**Experimental design and statistics**<br><br>Full details of the experimental design and statistical methods used should be given in the Methods section, as detailed in our Minimum Standards Reporting Checklist. Information essential to interpreting the data presented should be made available in the figure legends.<br><br>Have you included all the information requested in your manuscript?	Yes
**Resources**	Yes

Powered by Editorial Manager® and ProduXion Manager® from Aries Systems Corporation

A description of all resources used, including antibodies, cell lines, animals and software tools, with enough information to allow them to be uniquely identified, should be included in the Methods section. Authors are strongly encouraged to cite Research Resource Identifiers (RRIDs) for antibodies, model organisms and tools, where possible.<br><br>Have you included the information requested as detailed in our Minimum Standards Reporting Checklist?	
**Availability of data and materials**<br><br>All datasets and code on which the conclusions of the paper rely must be either included in your submission or deposited in publicly available repositories (where available and ethically appropriate), referencing such data using a unique identifier in the references and in the "Availability of Data and Materials" section of your manuscript.<br><br>Have you have met the above requirement as detailed in our Minimum Standards Reporting Checklist?	Yes
GigaScience has policies and guidelines in place for the use of generative AI-writing tools such as ChatGPT. If you have used such writing tools to assist with writing the manuscript this must be declared and cited in the text. Authors should not list AI-writing tools and other AI-assisted technologies as an author or co-author and should acknowledge that they are fully responsible for text generated or refined by AI-writing tools.<p><br><br>A summary of use (particularly in the introduction or among methods) needs to be included at the end of the paper, and the outputs should also be included as a supplementary file hosted in GigaDB or other open repositories. Please <a href=https://academic.oup.com/gigascience/pages/editorial_policies_and_reporting_	No

standards target="_new" > read our guidelines for more information. <p><br><br>By submitting to GigaScience, you are aware of the journal's AI-writing tools policy, and if you have declared use of such tools below, you have acknowledged this where appropriate in your manuscript and have made a summary of use and outputs available. <p><br><b>AI-assisted writing tools have been used in the preparation of this manuscript?	

Manuscript Click here to access/download;Manuscript;Manuscript_final.docx

ScopeViewer: A Browser-Based Solution for Visualizing Large Biological Images

Danni Luo[1], Sophie Robertson[2], Yuanchun Zhan[1], Ruichen Rong[1], Shidan Wang[1], Xi Jiang[1], Sen Yang[1], Suzette Palmer[1], Peiran Quan[1], Hiroaki Kanzaki[3], Yujin Hoshida[3], Liwei Jia[4], Qiwei Li[5], Guanghua Xiao[1,*], Xiaowei Zhan[1,*]

[1] Quantitative Biomedical Research Center, Peter O'Donnell Jr. School of Public Health, UT Southwestern Medical Center

[2] Paul Allen School of Computer Science & Engineering, University of Washington

[3] Department of Internal Medicine, UT Southwestern Medical Center

[4] Department of Pathology, UT Southwestern Medical Center

[5] Department of Mathematics Sciences, University of Texas at Dallas.

*To whom correspondence should be addressed.

Xiaowei.Zhan@UTSouthwestern.edu, Guanghua.Xiao@UTSouthwestern.edu

Abstract

Background

Spatial transcriptomics (ST) enables a high-resolution interrogation of molecular characteristics within specific spatial contexts and tissue morphology. Despite its potential, visualization of ST data is a challenging task due to the complexities in handling, sharing, and visualizing large image datasets together with molecular information.

Results

We introduce ScopeViewer, a browser-based software designed to overcome these challenges. ScopeViewer offers the following functionalities: (1) It visualizes large image data and associated annotations at various zoom levels, allowing for intricate exploration of the data; (2) It enables dual interactive viewing of the original images along with their annotations, providing a comprehensive understanding of the context; (3) It displays spatial molecular features with optimized bandwidth, ensuring a smooth user experience; and (4) It bolsters data security by circumventing data transfers.

Conclusions and Discussions

ScopeViewer offers the research community a convenient, powerful, and secure software for high-resolution images including pathology images and spatial transcriptomics. It serves as an open-source platform for imaging-based research. Future enhancements and new features will be shared on GitHub by the creators and are open for contributions from other researchers. ScopeViewer is freely available on the website at: https://cdc.biohpc.swmed.edu/scopeviewer.

1 Introduction

Spatial transcriptomics (ST) technologies have made significant advancements in recent years [1]. ST techniques offer high-resolution transcriptome measurements with spatial information within tissues, thereby opening new avenues for understanding cellular and molecular spatial distributions [2], and their associated links to diseases [3]. Recent computational advances have focused on improving spatial transcriptomics analysis through smoothing, spatial-domain identification, and graph-based representation learning approaches, including EAGS [4], Siamese graph autoencoders [5], and graph-attention autoencoder frameworks [6]. A typical ST dataset pairs with high-resolution images (e.g., H&E pathology slides), often comprising millions of pixels. This facilitates a dual visualization of cellular and tissue structures alongside quantitative molecular features, including gene expression and protein abundance. Examining molecular characteristics within spatial and morphological contexts could pave the way for new biological discoveries. A comprehensive tool for visualizing ST data will streamline data exploration and analysis, aiding researchers in comprehending molecular features within specific biological contexts.

Working with high-resolution tissue images and ST data introduces significant challenges due to the huge volume of these datasets. Consider a standard pathology image of 20,000 by 20,000 pixels, 0.5 microns per pixel, which can amass a file size of approximately one gigabyte. This large size complicates both the image's transfer and visualization, often requiring specialized software tools. Moreover, there is a high degree of complexity inherent in visualizing high-dimensional molecular features alongside the intricate cell and tissue structures. As a result, many software packages [7-9] currently require specific

preprocessing steps to display molecular details alongside high-resolution tissue images simultaneously. Further complications arise from software interfaces that require tedious manual input from users to toggle the visibility of data layers. A more streamlined solution is a synchronized dual-view approach, which addresses the limitations of single-view interfaces where molecular overlays often obscure underlying tissue morphology. This would allow users to see the data layer in one view, while simultaneously hiding it in another, with synchronized panning and zooming capabilities. Lastly, the ability for researchers to explore ST data on their own systems, without uploading or sharing data externally, is an important consideration. This not only bolsters data security but also enhances user accessibility. To address these prevalent challenges, we developed ScopeViewer, a browser-based visualization software, available online [10]. A docker image is also freely available online[11].

2 Methods and Results

ScopeViewer operates as a web application, requiring nothing more than a web browser for its execution. It was designed using ReactJS JavaScript framework. To utilize ScopeViewer, users simply navigate to the website and input the image information and ST data from a local path, using the JSON syntax. ScopeViewer generates an interactive user interface directly within the web browser. The platform's design leverages the versatility of web browsers, thereby eliminating the need for users to install specific software on their hardware.

2.1 Support for multiple imaging formats

When conducting pathology image analysis or exploring ST data, it is crucial to view the image at varying magnification levels. Additionally, users often need to overlay various sources for annotations. These might include (1) tissues from disparate anatomical locations; and (2) spatial spots generated by the 10x platform. To accommodate these needs, ScopeViewer incorporates the widely used deep zoom format (a Microsoft-maintained XML specification for viewing large images) and the advanced OpenSeaDragon platform in DZI, SVS, and TIFF formats. ScopeViewer's functionality extends beyond displaying multiple layers of large images at different magnification levels. It also supports dual views, a feature that enables side-by-side synchronized display (**Supplementary Fig. 1**). Additionally, ScopeViewer accommodates standard geometric annotations of various shapes, including lines, rectangles, ellipses, and polygons, through the integration of the Annotorious layer. These annotations can be manually created by human experts, such as pathologists, or generated automatically by AI software, like the HD-Yolo segmentation model [12]. The annotation data must be formatted in JSON, and users can conveniently specify the path to these layers within ScopeViewer's online JSON editor, which provides instant feedback for any syntax errors. By seamlessly visualizing these diverse annotations, ScopeViewer facilitates biological spatial pattern exploration and hypothesis generation.

2.2 Reduction of data transfer using a transpiled SQLite module

ST generates both high-resolution tissue image data and high-dimensional spatial molecular data, resulting in large datasets that are difficult to browse over the Internet. The process of transferring and processing such extensive ST data can be time-

consuming and challenging. To overcome this obstacle, we incorporated a tailored SQLite database implementation that offers two key features: (1) a WebAssembly version of SQLite. This was transpiled from its original C codes and provides high execution speed within the browser, which significantly enhances performance [13, 14]. (2) It has the capacity to fetch expression quantities from the SQLite database through HTTP Byte-Range headers. This functionality minimizes data transfer from the SQLite Virtual File System (VFS), making it more efficient. As a result of these optimizations, the webpage size is reduced from 180M (original molecular data) to 17.2M without cache, or 6.5M with the cache. This inventive approach simplifies the visualization of ST data within browser-based applications, enhancing user experience and enabling more effective research analysis.

2.3 Application: a breast cancer data from the 10X Visium platform

We demonstrate the use of ScopeViewer through a 10X Visium breast cancer FFPE sample [15]. To analyze the cell types and spatial distribution, we applied HD-Yolo, a deep-learning cell segmentation and classification algorithm on the whole slide image [12]. The image file measures 25,233 x 27,452 pixels and is 143M in size. Both the original image and its annotations are stored in DZI formats and deployed as the default ScopeViewer Demo to showcase three key features: (1) The original H&E slides are displayed on the left, while the algorithm-annotated image appears on the right within a synchronized interface (**Fig. 1A**). At the highest magnification level, tumor nuclei, necrosis, red blood cells, and stroma cells are annotated in green, cyan, magenta, and red, respectively. (2) The molecular transcriptome data (the spots) can be overlaid on the image (**Fig. 1B**). This shows that the cancer biomarker gene FASN is expressed highly

in the tumor region, clustering with tumor cells [16]. (3) No genomic data are transferred to the ScopeViewer web server, as data exchanges occur solely between the browser and the data server (**Fig. 1C**). This means the ScopeViewer web server instructs the user's browser to retrieve and display relevant information, without accessing potentially sensitive data, as its website does not communicate with the data server.

2.4 Application: a liver data from the 10X Visium platform

To further demonstrate the versatility of ScopeViewer across different tissue types and biological contexts, we applied it to a liver Visium dataset, also accessible under the "Examples" tab on the ScopeViewer website. We fixed surgically resected clinical liver tissues in 10% formalin and embedded in paraffin blocks. Five-μm-thick tissue section was used for sequencing library preparation using the Visium FFPE Spatial Gene Expression kit (10x Genomics) and sequenced with the NextSeq 500 system (Illumina) to generate genome-wide transcriptome profile for each circular region called "spot" (55 μm in diameter) on the tissue section. Raw data were preprocessed using the Space Ranger software ver. 1.3.0 (10x Genomics) based on reference genome (hg38). The spots with > 200 unique molecular identifier (UMI) counts were retained. Genes with a total UMI count < 100 across all spots, expressed in < 5 spots, and hemoglobin-related genes were excluded. We used ScopeViewer enables spatial visualization of pathogenic molecular dysregulations in stromal tissue in diseased liver. By using archived fixed surgical liver tissue affected with metabolic dysfunction-associated steatotic liver disease (MASLD), we performed Visium Spatial Transcriptome profiling. In MASLD liver, stromal tissue called portal tract is the major site of chronic inflammation and fibrogenesis that drive disease progression toward organ failure and cancer development [17]. In 11 out of

17 portal tracts with deposition of more fibrous tissues and/or lymphocyte infiltration, overexpression of markers of activated myofibroblasts (*ACTA2* and *COL3A1*), the major driver of liver fibrogenesis [18], and/or plasma/B cells (*JCHAIN*), the major driver of pre-portal hepatic injury [19], are clearly visualized (**Fig. 2**). This example demonstrates utility of ScopeViewer as a convenient alternative to laborious and technically often challenging immuno-staining to visualize spatial heterogeneity in pathogenic molecular dysregulations from H&E image and spatial transcriptome profiling data.

3 Conclusion and Discussion

ScopeViewer is a general, feature-rich, cloud-based, and secure tool for visualizing large biological images including ST datasets and H&E pathology slides. It specializes in secure, client-side rendering of large-scale spatial transcriptomics data without the need to upload genomic information, a feature not commonly found in other tools. Compared to existing tools such as Vitessce[20], TissUUmaps[21], CZ Cellxgene[22], Cytomine[7], Napari[9], and Giotto[8], ScopeViewer offers a distinct combination of features: secure client-side rendering that avoids genomic data transfer to external servers, dual synchronized views optimized for pathology workflows, and browser-native SQLite support enabling efficient retrieval of large spatial transcriptomics datasets without requiring server-side infrastructure. A detailed feature-by-feature comparison is provided in Supplementary Table 2. Furthermore, to our knowledge, ScopeViewer is among the first tools to support the browser native SQLite data format, enabling efficient retrieval of spatial data and flexible extension to general ST technology. This feature is enhanced by SQLite's built-in R-tree spatial index. Lastly,

ScopeViewer is optimized for user interaction and offers dual-view synchronized visualization for conveniently exploring raw images, histology and molecular annotations, providing a novel enhancement for detailed tissue inspection. We envision that it will be a valuable resource for data exploration and sharing within the wider research community.

Online resource

We used Javascript and nodejs backend to implement the ScopeViewer. We provided the online resource source [10]and prepared Docker images at DockerHub[11]. Additionally, we provide examples that include single pathology images with hierarchical annotations, a list of pathology images, and ST data from the 10X Visium platform. ScopeViewer can also be customized to visualize new datasets for future digital pathology or ST studies. The codes are licensed under GNU General Public License v3.0.

Availability of source code and requirements

Project name: ScopeViewer

Project home page: https://cdc.biohpc.swmed.edu/scopeviewer

Operating system(s): Web browser

Programming language: Javascript (nodejs version 16)

Other requirements: N/A

License: GNU General Public License v3.0

Data availability

Spatial transcriptome profiling dataset is publicly available: breast cancer dataset is from 10X Genomics public datasets, and liver disease dataset was generated in-house and has been deposited to the NCBI GEO (accession number: GSE278621). Additional technical details and demonstration of ScopeViewer functions are available in **Supplementary Texts** and **Figures**. To address the complexities of manual JSON configuration, we provide a Python helper script (database.py) that automates the creation of these JSON files, significantly lowering the barrier for users without prior coding or bioinformatics experience. Source codes to facilitate users to prepare ST data can be found at [23]. Comprehensive user guides on how to prepare data can also be found at [24]. Furthermore, a step-by-step video tutorial demonstrating this local setup and auto-configuration process is available in [25] . Alternatively, a Docker image of ScopeViewer is available online [10]. We also provide user support through GitHub issues to address researchers' specific needs.

List of abbreviations

H&E: Hematoxylin and Eosin

ST: Spatial transcriptomics

MASLD: Metabolic dysfunction-associated steatotic liver disease.

Ethic

Not applicable.

Consent for publication

10X Visium breast cancer FFPE dataset is provided by 10X genomics which grants the researchers non-commercial access permission.

Competing Interests

No competing interest was reported by any author.

Funding

This study is partially supported by the National Institutes of Health (U01AI169298 [XZ], R01HG011035 [XZ], R01GM140012 [GX], R01GM115473 [GX], R01DE030656 [GX], 1U01CA249245 [GX]) and the Cancer Prevention and Research Institute of Texas (CPRIT RP230330 [GX]).

Author's Contributions

D.L., S.R., and Y. Z. performed the experiment and wrote software. R.R., S, W., S. Y., S. P., L. J., and Q.L. provided resources and helpful discussions. D.L., G.X. and X.Z. designed the experiment, performed data analysis, and wrote the manuscript.

Acknowledgments

We thank Ismael Villanueva-Miranda, Jonathan Wang, Fangjiang Wu, Shengjie Yang, and Qinbo Zhou for their contribution to the software development and/or comments on the manuscript.

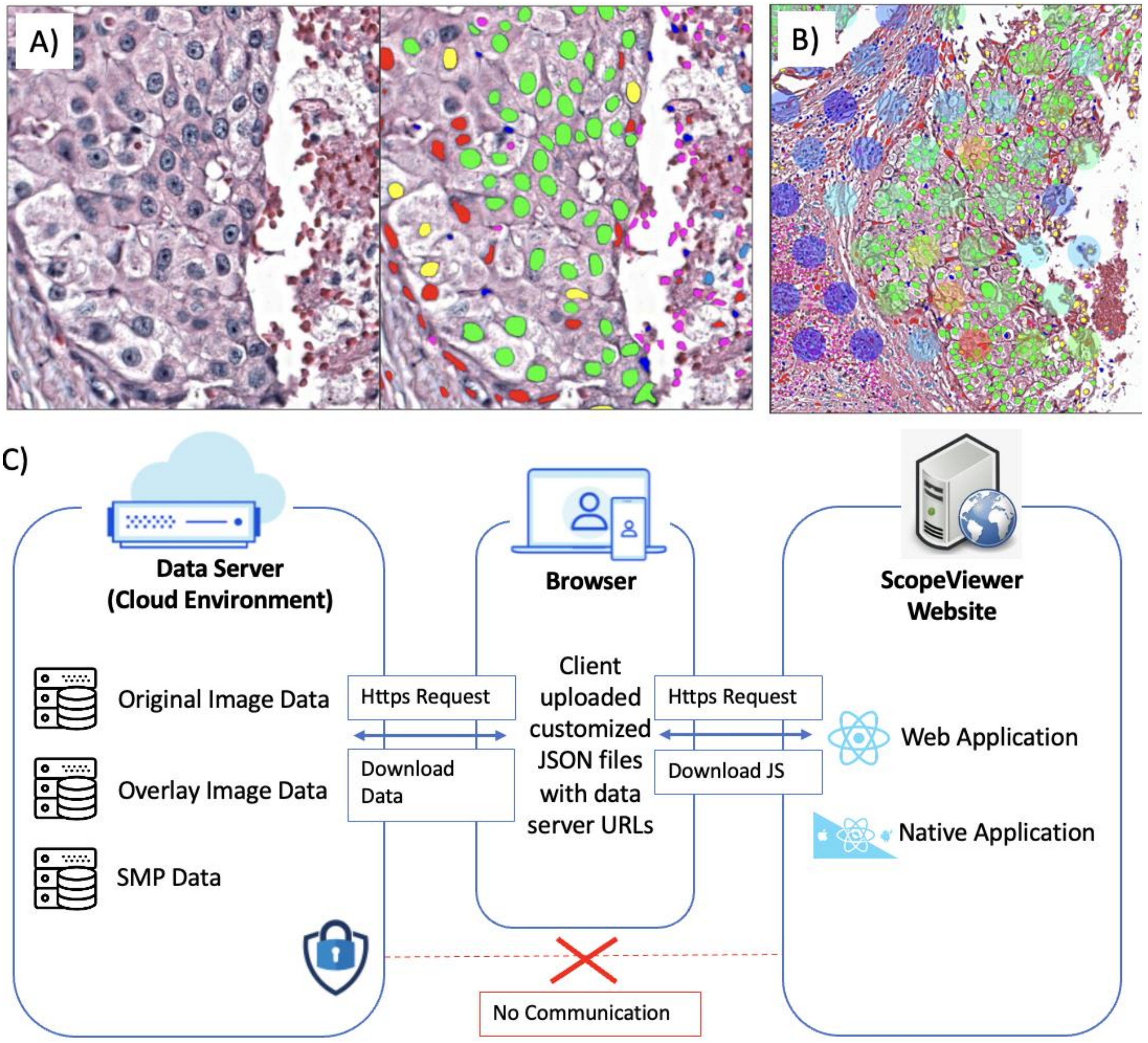

Figure 1: ScopeViewer for visualization ST data.
(A) synchronized views for H&E pathology image and AI facilitated cell segmentation; (B) efficiently overlaying gene expression features (here shown *FASN*, a breast cancer biomarker gene); (C) visualization will not leak genomic data to the ScopeViewer webserver.

1

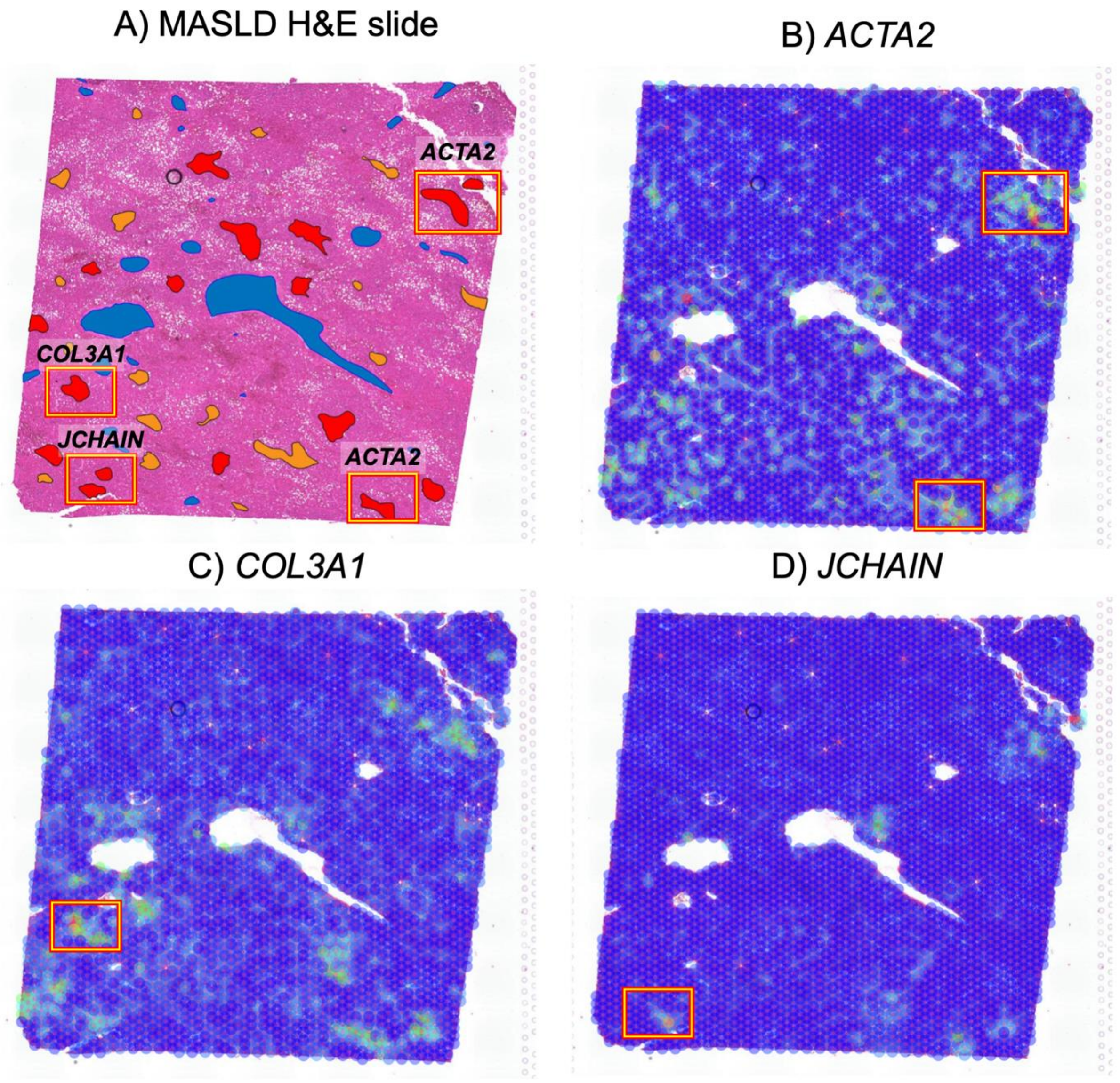

Figure 2. Heterogeneous pattern of spatial transcriptomics in MASLD.
(**A**) Liver H&E slides with pathology annotations (blue: central vein; red: inflammatory/fibrotic portal tract; orange: non inflammatory/fibrotic portal tract). Overexpression of activated myofibroblasts marker genes, *ACTA2* (**B**) and *COL3A1*(**C**), and liver fibrogenesis signatures (**D**) overlaps with inflammatory/fibrotic regions, demonstrating heterogeneous biologic pattern. MASLD: metabolic dysfunction-associated steatotic liver disease.

Reference

1. Zhang M, Sheffield T, Zhan X, Li Q, Yang DM, Wang Y, et al. Spatial molecular profiling: platforms, applications and analysis tools. Brief Bioinform. 2021;22 3 doi:10.1093/bib/bbaa145.
2. Crosetto N, Bienko M and van Oudenaarden A. Spatially resolved transcriptomics and beyond. Nat Rev Genet. 2015;16 1:57–66. doi:10.1038/nrg3832.
3. Shah S, Takei Y, Zhou W, Lubeck E, Yun J, Eng CL, et al. Dynamics and Spatial Genomics of the Nascent Transcriptome by Intron seqFISH. Cell. 2018;174 2:363–76 e16. doi:10.1016/j.cell.2018.05.035.
4. Lv T, Zhang Y, Li M, Kang Q, Fang S, Zhang Y, et al. EAGS: efficient and adaptive Gaussian smoothing applied to high-resolved spatial transcriptomics. GigaScience. 2024;13 1 doi:10.1093/gigascience/giad097.
5. Cao L, Yang C, Hu L, Jiang W, Ren Y, Xia T, et al. Deciphering spatial domains from spatially resolved transcriptomics with Siamese graph autoencoder. GigaScience. 2024;13 1 doi:10.1093/gigascience/giae003.
6. Zhou L, Peng X, Chen M, He X, Tian G, Yang J, et al. Unveiling patterns in spatial transcriptomics data: a novel approach utilizing graph attention autoencoder and multiscale deep subspace clustering network. GigaScience. 2025;14 doi:10.1093/gigascience/giae103.
7. Maree R, Rollus L, Stevens B, Hoyoux R, Louppe G, Vandaele R, et al. Collaborative analysis of multi-gigapixel imaging data using Cytomine. Bioinformatics. 2016;32 9:1395–401. doi:10.1093/bioinformatics/btw013.

8. Dries R, Zhu Q, Dong R, Eng CL, Li H, Liu K, et al. Giotto: a toolbox for integrative analysis and visualization of spatial expression data. Genome biology. 2021;22 1:78. doi:10.1186/s13059-021-02286-2.

9. Chiu C-L and Clack N. Napari: a Python multi-dimensional image viewer platform for the research community. Microscopy and Microanalysis. 2022;28 S1:1576–7.

10. Luo D, Robertson S, Zhan Y, Rong R, Wang S, Jiang X, et al.: ScopeViewer Website. https://cdc.biohpc.swmed.edu/scopeviewer (2026).

11. Luo D, Robertson S, Zhan Y, Rong R, Wang S, Jiang X, et al.: ScopeViewer Docker Image. https://hub.docker.com/r/utsw1qbrc/scopeviewer. (2026).

12. Rong R, Sheng H, Jin KW, Wu F, Luo D, Wen Z, et al. A Deep Learning Approach for Histology-Based Nucleus Segmentation and Tumor Microenvironment Characterization. Mod Pathol. 2023;36 8:100196. doi:10.1016/j.modpat.2023.100196.

13. Andrés BF and Pérez M. Transpiler-based architecture for multi-platform web applications. In: *2017 IEEE Second Ecuador Technical Chapters Meeting (ETCM)* 16–20 Oct. 2017 2017, pp.1–6.

14. Rossberg A. Webassembly specification. WebAssembly Community Group. 2021.

15. Janesick A, Shelansky R, Gottscho AD, Wagner F, Rouault M, Beliakoff G, et al. High resolution mapping of the breast cancer tumor microenvironment using integrated single cell, spatial and in situ analysis of FFPE tissue. bioRxiv. 2022:2022.10.06.510405. doi:10.1101/2022.10.06.510405.

16. He B, Bergenstrahle L, Stenbeck L, Abid A, Andersson A, Borg A, et al. Integrating spatial gene expression and breast tumour morphology via deep learning. Nat Biomed Eng. 2020;4 8:827–34. doi:10.1038/s41551-020-0578-x.

17. Suzuki H, Fujiwara N, Singal AG, Baumert TF, Chung RT, Kawaguchi T, et al. Prevention of liver cancer in the era of next-generation antivirals and obesity epidemic. Hepatology. 2025; doi:10.1097/HEP.0000000000001227.

18. Krenkel O, Hundertmark J, Ritz TP, Weiskirchen R and Tacke F. Single Cell RNA Sequencing Identifies Subsets of Hepatic Stellate Cells and Myofibroblasts in Liver Fibrosis. Cells. 2019;8 5 doi:10.3390/cells8050503.

19. Dhingra S, Mahadik JD, Tarabishy Y, May SB and Vierling JM. Prevalence and clinical significance of portal inflammation, portal plasma cells, interface hepatitis and biliary injury in liver biopsies from patients with non-alcoholic steatohepatitis. Pathology. 2022;54 6:686–93. doi:10.1016/j.pathol.2022.01.009.

20. Keller MS, Gold I, McCallum C, Manz T, Kharchenko PV and Gehlenborg N. Vitessce: integrative visualization of multimodal and spatially resolved single-cell data. Nature Methods. 2024;21:1778–86. doi:10.1038/s41592-024-02436-x.

21. Pielawski N, Andersson A, Avenel C, Behanova A, Chelebian E, Klemm A, et al. TissUUmaps 3: improvements in interactive visualization, exploration, and quality assessment of large-scale spatial omics data. Heliyon. 2023;9 5:e15306. doi:10.1016/j.heliyon.2023.e15306.

22. Program CZICS, Abdulla S, Aevermann B, Megill C, Mani A, Cool J, et al. CZ CELLxGENE Discover: a single-cell data platform for scalable exploration,

analysis and modeling of aggregated data. Nucleic Acids Research. 2025;53 D1:D886–D95. doi:10.1093/nar/gkae1142.

23. Luo D, Robertson S, Zhan Y, Rong R, Wang S, Jiang X, et al.: ScopeViewer Database for Spatial Transcriptomics. https://cdc.biohpc.swmed.edu/scopeviewer/database.py (2026).

24. Luo D, Robertson S, Zhan Y, Rong R, Wang S, Jiang X, et al.: ScopeViewer Data Preparation Script. https://cdc.biohpc.swmed.edu/scopeviewer/prepareData (2026).

25. Luo D, Robertson S, Zhan Y, Rong R, Wang S, Jiang X, et al.: ScopeViewer Tutorial. https://youtu.be/0edwi6C_eM8 (2026).

Click here to access/download
Supplementary Material
Supplementary Materials final v2.docx

Click here to access/download
Supplementary Material
Responding to Reviewer-Final.docx

Cover letter
Click here to access/download;Personal Cover;CoverLetter R2.docx

UT SOUTHWESTERN MEDICAL CENTER

March 31, 2026

GigaScience

Dear Editor:

We are submitting a revised manuscript entitled "ScopeViewer: A Browser-Based Solution for Visualizing Large Biological Images" by Luo et al., which we respectfully ask you to consider for publication in GigaScience. This submission represents an extensive revision following previous communication regarding manuscript ID GIGA-D-23-00318. The manuscript presents original research that has not been published or publicly presented elsewhere.

Based on the constructive comments from the reviewers, this revision addresses their specific feedback by clarifying ScopeViewer's unique contributions, improving usability and reproducibility, and strengthening the biological context. The revised manuscript emphasizes the synchronized dual-view visualization and more carefully positions ScopeViewer relative to six widely used tools through a systematic comparative analysis. Usability has been enhanced through automated helper scripts, step-by-step video tutorials, improved documentation, a Docker image for offline use, and a fully downloadable example dataset. Reproducibility concerns were addressed by releasing the complete source code and providing a stable Docker deployment. Finally, we expanded the biological scope with an additional liver MASLD application, added new figures and interpretations, clarified annotation and platform compatibility, and updated multiple manuscript sections to reflect these changes in a more balanced and rigorous manner.

All co-authors have reviewed the submitted manuscript and approved the findings and conclusions. Please feel free to contact us if you require any additional information.
Thank you for your consideration of our work.

Sincerely,

Xiaowei Zhan

Xiaowei Zhan
