# Supplementary material for: ScopeViewer: a browser-based solution for visualizing large biological images
Source: Gigascience. 2026 Jun 22;15:giag074. doi: 10.1093/gigascience/giag074 (PMC13369961; doi:10.1093/gigascience/giag074)
Supplement: giag074_Reviewer_1_Report_Original_Submission — Reviewer 1 -- 10/12/2025 [file giag074_reviewer_1_report_original_submission.pdf]

Reviewer Report

Title: ScopeViewer: A Browser-Based Solution for Visualizing Large Biological Images

Version: Original Submission **Date:** 10/12/2025

Reviewer name: Reviewer 1

Reviewer Comments to Author:

ScopeViewer runs within a browser, eliminating the hassle of configuring specific Python or other environments required by similar tools. This browser-based cross-platform tool offers superior compatibility. It enables the dual-view comparison of annotated images and raw HE images, offering greater convenience than other tools. Additionally, ST files are typically large, and this tool significantly mitigates slow transfer issues for large files. Overall this is a useful tool that will be very helpful to the ST research community. However, this manuscript still has some issues that need to be addressed.

1. Benchmarking against other tools shows that, although ScopeViewer offers the unique advantage of dual synchronised views, its overall performance is no better than that of other available tools. This distinctive strength should also be explicitly highlighted in the main text.
2. While the web interface slightly lowers the barrier to entry for users, it may not offer revolutionary convenience. Many web-based tools allow users to drag and drop files or make simple selections, while ScopeViewer requires users to create a dedicated JSON file, which may be challenging for those with limited bioinformatics experience.
3. I noticed some open-source code was missing. GitHub only hosts a subset of the project sources, not the complete repository.
4. The manuscript claims that "ScopeViewer is the only tool that supports the browser native SQLite data format". Is there any literature or evidence substantiating this "uniqueness"?
5. The application example provided in the article uses breast cancer data from the 10x Genomics platform only. Using only this single example, without data from other cancer types or measurement platforms, presents certain limitations.

Methods

Are the methods appropriate to the aims of the study, are they well described, and are necessary controls included? Choose an item.

Conclusions

Are the conclusions adequately supported by the data shown? Choose an item.

Reporting Standards

Does the manuscript adhere to the journal’s guidelines on minimum standards of reporting? Choose an item.

Choose an item.

Statistics

Are you able to assess all statistics in the manuscript, including the appropriateness of statistical tests used? Choose an item.

Quality of Written English

Please indicate the quality of language in the manuscript: Choose an item.

Declaration of Competing Interests

Please complete a declaration of competing interests, considering the following questions:

- Have you in the past five years received reimbursements, fees, funding, or salary from an organisation that may in any way gain or lose financially from the publication of this manuscript, either now or in the future?
- Do you hold any stocks or shares in an organisation that may in any way gain or lose financially from the publication of this manuscript, either now or in the future?
- Do you hold or are you currently applying for any patents relating to the content of the manuscript?
- Have you received reimbursements, fees, funding, or salary from an organization that holds or has applied for patents relating to the content of the manuscript?
- Do you have any other financial competing interests?
- Do you have any non-financial competing interests in relation to this paper?

If you can answer no to all of the above, write 'I declare that I have no competing interests' below. If your reply is yes to any, please give details below.

I declare that I have no competing interests

I agree to the open peer review policy of the journal. I understand that my name will be included on my report to the authors and, if the manuscript is accepted for publication, my named report including any attachments I upload will be posted on the website along with the authors' responses. I agree for my report to be made available under an Open Access Creative Commons CC-BY license (http://creativecommons.org/licenses/by/4.0/). I understand that any comments which I do not wish to be included in my named report can be included as confidential comments to the editors, which will not be published.

Choose an item.

To further support our reviewers, we have joined with Publons, where you can gain additional credit to further highlight your hard work (see: https://publons.com/journal/530/gigascience). On publication of this paper, your review will be automatically added to Publons, you can then choose whether or not to claim your Publons credit. I understand this statement.

Yes Choose an item.
