# Supplementary material for: ScopeViewer: a browser-based solution for visualizing large biological images
Source: Gigascience. 2026 Jun 22;15:giag074. doi: 10.1093/gigascience/giag074 (PMC13369961; doi:10.1093/gigascience/giag074)
Supplement: giag074_Reviewer_3_Report_Original_Submission — Reviewer 3 -- 11/3/2025 [file giag074_reviewer_3_report_original_submission.pdf]

Reviewer Report

Title: ScopeViewer: A Browser-Based Solution for Visualizing Large Biological Images

Version: Original Submission **Date:** 11/3/2025

Reviewer name: Reviewer 3

Reviewer Comments to Author:

This manuscript introduces ScopeViewer, a browser-based platform for visualizing large biological images, including spatial transcriptomics and pathology data. The topic is timely, and the software appears to be a more accessible and technically advanced implementation compared with existing visualization tools such as Loupe Browser or CellXGene Viewer. The work addresses an important technical challenge and presents a promising browser-based solution.

However, to make the tool truly useful to the research community and to clearly distinguish it from existing tools, the manuscript still lacks essential information in two main aspects: biological relevance and usability for end users.

Major Comments

1. Biological Interpretation

The manuscript should more clearly demonstrate how ScopeViewer contributes to biological discovery and interpretation. The current example focuses on cell segmentation and classification results, which are valuable but represent only a modest improvement over existing tools.

The authors should elaborate on what additional types of biological information can be visualized or analyzed within the tool. For example:

* What kinds of annotations are supported (e.g., cell boundaries, morphology, clusters, tissue regions, spot-level measurements)?
* How are these annotations generated, formatted, and loaded into ScopeViewer?
* How do these annotations facilitate the integration of molecular and cellular features for biological interpretation?

I recommend providing an additional example with more specific biological questions, either in the supplementary materials or on the project website to demonstrate more diverse and distinctive applications of ScopeViewer. This would better highlight its scientific and practical advantages.

2. Ease of Use and Reproducibility

The usability of ScopeViewer could be enhanced through more detailed, step-by-step guidance. I strongly recommend including:

* A reproducible and downloadable example dataset (with corresponding annotations) so users can easily replicate the workflow and apply it to their own data.
* A clear data preparation guide describing how users can prepare or convert their datasets (for example, a typical 10x Visium dataset from a public repository), including what kinds of annotations can be generated and how they can be converted into the required JSON format.

Providing this information in the documentation or supplementary materials would substantially improve accessibility, reproducibility, and community adoption.

Minor Comment
It would be helpful to clarify whether ScopeViewer supports newer high-resolution spatial omics platforms such as Visium HD, or Xenium, and to specify the extent to which users can visualize or explore data from these technologies.
Overall Evaluation:
ScopeViewer is a technically solid and promising tool with clear potential for broad application. Enhancing the manuscript with richer biological examples and comprehensive user guidance would significantly improve its impact and usability for the research community.

Methods

Are the methods appropriate to the aims of the study, are they well described, and are necessary controls included? Choose an item.

Conclusions

Are the conclusions adequately supported by the data shown? Choose an item.

Reporting Standards

Does the manuscript adhere to the journal's guidelines on minimum standards of reporting? Choose an item.

Choose an item.

Statistics

Are you able to assess all statistics in the manuscript, including the appropriateness of statistical tests used? Choose an item.

Quality of Written English

Please indicate the quality of language in the manuscript: Choose an item.

Declaration of Competing Interests

Please complete a declaration of competing interests, considering the following questions:

- Have you in the past five years received reimbursements, fees, funding, or salary from an organisation that may in any way gain or lose financially from the publication of this manuscript, either now or in the future?
- Do you hold any stocks or shares in an organisation that may in any way gain or lose financially from the publication of this manuscript, either now or in the future?

- Do you hold or are you currently applying for any patents relating to the content of the manuscript?
- Have you received reimbursements, fees, funding, or salary from an organization that holds or has applied for patents relating to the content of the manuscript?
- Do you have any other financial competing interests?
- Do you have any non-financial competing interests in relation to this paper?

If you can answer no to all of the above, write 'I declare that I have no competing interests' below. If your reply is yes to any, please give details below.

I declare that I have no competing interests

I agree to the open peer review policy of the journal. I understand that my name will be included on my report to the authors and, if the manuscript is accepted for publication, my named report including any attachments I upload will be posted on the website along with the authors' responses. I agree for my report to be made available under an Open Access Creative Commons CC-BY license (http://creativecommons.org/licenses/by/4.0/). I understand that any comments which I do not wish to be included in my named report can be included as confidential comments to the editors, which will not be published.

Choose an item.

To further support our reviewers, we have joined with Publons, where you can gain additional credit to further highlight your hard work (see: https://publons.com/journal/530/gigascience). On publication of this paper, your review will be automatically added to Publons, you can then choose whether or not to claim your Publons credit. I understand this statement.

Yes Choose an item.
